# Proprioception in Action: A Matter of Ecological and Social Interaction

**DOI:** 10.3389/fpsyg.2020.569403

**Published:** 2021-01-14

**Authors:** Ximena González-Grandón, Andrea Falcón-Cortés, Gabriel Ramos-Fernández

**Affiliations:** ^1^Departamento de Educación, Universidad Iberoamericana, Ciudad de México, Mexico; ^2^Facultad de Medicina, Universidad Nacional Autónoma de México, Ciudad de México, Mexico; ^3^Insituto de Filosofía y Ciencias de la Complejidad IFICC-Chile, Santiago, Chile; ^4^Instituto de Ciencias Físicas, Universidad Nacional Autónoma de México, Cuernavaca, Mexico; ^5^Instituto de Investigaciones en Matemáticas Aplicadas y en Sistemas, Universidad Nacional Autónoma de México, Ciudad de México, Mexico; ^6^Centro de Ciencias de la Complejidad, Universidad Nacional Autónoma de México, Ciudad de México, Mexico

**Keywords:** enactive cognition, sensorimotor theory, perception-action-coupling, ecological self, social cognition, agent-based models, kinesthetic phenomenology

## Abstract

The aim of this paper is to provide a theoretical and formal framework to understand how the proprioceptive and kinesthetic system learns about body position and possibilities for movement in ongoing action and interaction. Whereas most weak embodiment accounts of proprioception focus on positionalist descriptions or on its role as a source of parameters for internal motor control, we argue that these aspects are insufficient to understand how proprioception is integrated into an active organized system in continuous and dynamic interaction with the environment. Our strong embodiment thesis is that one of the main theoretical principles to understand proprioception, as a perceptual experience within concrete situations, is the coupling with kinesthesia and its relational constitution—self, ecological, and social. In our view, these aspects are underdeveloped in current accounts, and an enactive sensorimotor theory enriched with phenomenological descriptions may provide an alternative path toward explaining this skilled experience. Following O'Regan and Noë (2001) sensorimotor contingencies conceptualization, we introduce three distinct notions of proprioceptive kinesthetic-sensorimotor contingencies (PK-SMCs), which we describe conceptually and formally considering three varieties of perceptual experience in action: PK-SMCs-self, PK-SMCs-self-environment, and PK-SMC-self-other. As a proof of concept of our proposal, we developed a minimal PK model to discuss these elements in detail and show their explanatory value as important guides to understand the proprioceptive/kinesthetic system. Finally, we also highlight that there is an opportunity to develop enactive sensorimotor theory in new directions, creating a bridge between the varieties of experiences of oneself and learning skills.

## Introduction

Suppose you have just woken up and immediately you feel the presence of your body; then, or maybe at the same time, you feel a body that is not yours cuddling you and perhaps also the sheets that do not cover your feet, leaving them uncovered. Your own body experience is subtly transformed with each focus of attention, as it takes on a distinctively ecological and social dimension. Both agents are sharing this proprioceptive and kinesthetic experience with each other. Can such embodied, ecological, and social interaction be part of an agent's proprioceptive perceptual experience?

In embodied accounts of proprioception, there are some disagreements about the explanatory role of the non-neural elements in real-time interaction. Alsmith and De Vignemont (2012, p. 1–13), for instance, propose a distinction between weak and strong approaches to body involvement. In the weak embodiment account, mental representations in bodily formats play a central role in action and perception, while moving bodies in interaction—a non-brain-bounded element—play a trivial one. These “B-formats” are associated with muscular sensation, as a physiological condition of the body (Goldman and de Vignemont, 2009), and become crucial when they are centrally represented in the brain and instantiated in internal models (Goldman, 2012). Strong embodiment accounts, in contrast, consider the whole body in its dynamical gestalt-like relations with physical and social environments as non-neural elements that play a strong causal and constitutive role in perception and action (Varela et al., 1991; O'Regan and Noë, 2001; Gallagher, 2017). Here, perception is a bodily experience intimately linked to skillful and effective embodied possibilities for action. Moreover, in these accounts, proprioception is better understood coupled with kinesthesia (as a proprioceptive-kinesthetic coupling or PK for short), a perceptual system that results from an active and ongoing coupling between feeling and performing.

Traditional accounts of proprioception place a special emphasis on the “self-perception” related to the body awareness of an agent's relative position in space. This is a central idea that can be found in weak embodiment approaches. Commonly, this positional sense description comes from Sherrington (1907) and his original conceptualization, in which the central nervous system (CNS) receives information about the spatial location of body parts and body segments to enable movement coordination. According to this, the experience of proprioception is described as a key source of spatial parameters for internal motor control at the level of the sensor: if an agent wants to put an earring into her earlobe, for example, she needs to wiggle her fingers around a bit to get it in and find the piercing hole. Here, a flexible transformation from proprioceptive afferent information about the position of the fingers is needed, for the capacity to estimate the appropriate set of motor commands required to achieve the desired outcome. In this model, however, experiencing one's body comes from verifying whether these estimations match or not in a controlled act, and the possibilities for variations are thus almost entirely determined.

The main objective of the present article is to introduce a strong embodiment account of proprioception based on O'Regan and Noë (2001) enactive sensorimotor theory of perception (ESMT) and Sheets-Johnstone (2019, 2020) kinesthetic phenomenology; as well as offer a formalization of this proposal following the work of Buhrmann et al. (2013) and Vicsek et al. (1995). This alternative account considers PK as a perceptual experience of spatiotemporal self-orientation in present action and interaction. On the one hand, from an enactive point of view—one that sees the perceiver as an active organism engaging with the ecological and social world—how the agent puts an earring into her earlobe depends on where her fingers are in relation to the rest of her body and where the piercing perforation is, how it feels, the previous experiences putting an earring, whether the surface where she is standing is flat or not, whether another agent is helping her, etc. This suggests that for the action to be effective, we not only need to perceive the objects on which we act or the state of the effector, such as the earring when inserted, but also the real-time PK experiences of the lived body whether dancing or walking.

In this view, a strong embodiment account of the proprioceptive perceptual experience should articulate, in operational and (if possible) formal terms, what these meaningful and skilled relations consist of. Here, we tackle this challenge by arguing that the PK perceptual experience is not only caused by some internal process in the brain—as a B-format representation or a specific somatosensorial cortex correlate—but rather that it is constituted by an organism's set of abilities to act during the ongoing affair of establishing meaningful relations with one's body and the world (O'Regan and Noë, 2001), that is, its proprioceptive-kinesthetic contingencies (PK-SMCs).

We propose that some dynamical self-oriented and relational features of the phenomenology of PK, resulting from coupling of perception and action, constitute the PK perceptual experience. Specifically, this is manifested in at least three different dimensions by the various degrees in which this experience occurs during a common episode of being present and bodily aware and ready to act: first, PK-SMCs-self that are related to the agent's own spatio-temporal self-orientation, in relation to other parts of one's body, and possibilities for action in present time; second, PK-self-ecological, which are those that arise from the agent's own embodied activity when interacting with the environment; and third, PK-self-other, which are those that arise from the agent's own activity when interacting with others. We will argue that these relational dimensions can be useful tools for explaining the PK-SMCs perceptual experience.

Finally, we illustrate the usefulness of these distinctions by applying them to the analysis of a model of minimal cognition of collective movement perception (following the work of Vicsek et al., 1995; Beer, 2003 and others). In this model, spatial and temporally organized behavior arise in agents with both skilled PK and non-skilled PK and in agents with any recourse to PK (deafferented agents) moving continuously inside a square. With this model, we achieve the dual purpose of testing the operational character of conceptual claims about PK perceptual experience from a strong embodiment account, and of bringing together ESMT and phenomenology while showing some limitations of the weak and current accounts.

## What Is Proprioceptive/Kinesthetic Coupling?

In order to have different opportunities of movement and to behave adequately in different environments, both known and unknown, an organism that recognizes itself separate from the environment has to master particular skills. The ability to recognize being in “the zero point of orientation” (Husserl, 1989) and being the origin of one's own movement, as a form of sensitivity to embodied actions, requires the concurring development of the skills to experience the spatio-temporal self-orientation, and the feeling of possibilities for action. In this section, we will argue that proprioception and kinesthesia (as a PK coupling) have a central role in the development of this ability (Gallagher, 2003; Gapenne, 2014). In further sections, we will see that PK is also relevant to engage successfully in ecological and social interactions.

From a physiological standpoint, proprioception encompasses information from specialized sensory mechanoreceptors primarily found in muscles, such as neuromuscular spindles or neurotendinous organs, but also in the joints, tendons, ligaments, articulatory capsules, vestibular apparatus, or skin. These receptors transduce mechanical events into neural signals (Proske and Gandevia, 2012). In fact, muscle spindles provide the central nervous system (CNS) with afferent information about the length and velocity of the muscle in which the spindles are embedded and their rate of change, contributing to joint position sense and postural control. Traditionally, this has been considered as the main source of proprioceptive feedback for spinal sensorimotor regulation and servo-control (Sherrington, 1907; Fourneret and Jeannerod, 1998; Hewett et al., 2002)^1^.

In this sense, proprioception is the perception of the relative positions of different body parts, where suitable proprioceptive sensors register joint angles and the activity of the effectors to which they are linked. These ideas are more aligned with the weak embodiment account. When trying to understand what the content of proprioceptive perceptual experience is, authors like Goldman (2012) or Goldman and de Vignemont (2009) have appealed to the existence of non-propositional B-formats. These are internal representations “associated with the physiological conditions of the body, such as pain, temperature, itch, muscular and visceral sensations, vasomotor activity, hunger, and thirst” (Goldman and de Vignemont, 2009, p. 156). Following these authors, B-formatted representations may originate peripherally and involve proprioceptive or kinesthetic information about the agent's own muscles. However, when represented centrally, they become genuinely B-formatted representations: “for example, codes associated with activations in somatosensory cortex and motor cortex” (Goldman, 2012, p. 74). When considering proprioception from this perspective, an implicit representationalist and brain-centered bias may emerge, where actual sensing and moving bodies play a marginal role. Indeed, this weak embodiment perspective restricts proprioception to the sensations about position produced by the static body and does not include the organization and the quality of the possibilities for movement from the proprioceptive self.

At this point, some accounts distinguish between proprioception and kinesthesia. For instance, human physiology has traditionally distinguished static sensations of one's joint positions (proprioception), from dynamic sensations, such as those that are sensitive to the rate of a specific movement (kinesthesia) (Kiefer et al., 2013). Indeed, kinesthesia was originally recognized as “the muscle sense,” the sense of actions of the limbs (the sense of one's own movement), or the perceived sensations of positions in a system of possible movements (Sherrington, 1918). In this article, rather than subsume kinesthesia to proprioception or vice versa, or propose a distinction between them, we follow Sheets-Johnstone (2019) and Gapenne (2014) to suggest that proprioception is necessarily coupled with kinesthesia and possibilities for action (Gapenne, 2010, 2014): an emergent form of organization between sensing the spatio-temporal self-orientated body and the possibilities for the performing body.

Closer to the strong embodiment perspective, we argue that proprioception separated from kinesthesia fails to do justice to the different levels of analysis on which organisms' perceptual experience can be described. In the next section, we argue that this coupling can be understood more precisely in an ecological context.

###  Ecological Laws in PK

As argued by several investigations, although perception and action are mediated by different processes and pathways, they are coupled by ecological laws that relate afferent variables to parameters of the action system to regulate behavior adaptively (Varela et al., 1991; Warren, 2006; Dayan et al., 2007; Gonzalez-Grandón and Froese, 2018). This is implied by the notion of perception-action coupling from an ecological standpoint, which is made explicit by Gibson (1977, p. 223) in the following passage: “We must perceive in order to move, but we must also move in order to perceive.” From this perspective, the perceptual prominence of vertebrate movement might come from these close interactions and regularities: the so-called ecological laws, such as attractors in the underlying dynamics between perception and action (Warren, 2006; without assuming predetermined or *a priori* cognitive or neural models; Dayan et al., 2007).

These ideas are a crucial background to the emergence of ESMT, an action-oriented perspective relying on enaction—putting into practice through action—where perceptual contingencies are intrinsically tied to specific movements. As Noë (2004, p. 2) states, perception is a “species of skillful bodily activity.” In the coupling case we are concerned with, these ecological laws would be related to proprioception and kinesthesia. Proprioceptive information is both generated by and reciprocally used to regulate kinesthetic possibilities for movement. By information, Gibson (1977) meant spatio-temporal proprioceptive patterns of joint, muscle, or skin deformation at a moving limb, that are lawfully related to properties of the perturbations of the environment or aspects of the possibilities for the action itself. We can elaborate on this notion in terms of perception-action coupling.

An illustrative example comes from motor development in infancy, where researchers have begun to entertain that perceptual and motor systems develop in interdependent trajectories. Thelen (1990) provides evidence that motor skill emerges in development as a dynamic and spontaneous process through recurrent perception-action loops where knowledge of the external world is integrated with knowledge of self-movement (continuous exploration of the infant's own body) as the body moves through a force field.

Findings from behavioral brain research also provide evidence for this perception-action coupling. Alaerts et al. (2007), by means of a tracking task, show that proprioception is subject to constraints from extrinsic and intrinsic reference frames that are continuously updated^2^.

Building upon these theoretical and empirical perspectives, we propose that PK is organizationally integrated as a coupled system, not restricted to the constant activation from deformations of the dynamic body to produce sensations about the position or the movements of the limbs (Sherrington, 1907; Fourneret and Jeannerod, 1998; Hewett et al., 2002). Thus, the central nervous system would not be unique in its capacity to control the wide variety of action-oriented abilities. Rather, these abilities would arise from a systemic regulation, including cortical and subcortical networks, effector organs, sensed environmental constraints, such as gravity and friction (Goodwin et al., 1972; Gapenne, 2014), as well as sensed social constraints, such as those related to social interaction. However, this organization in action remains ambiguous.

## Proprioception in Action as a Puzzle: Is an Internal Model the Missing Piece?

Most accounts in which proprioception seems to be coupled with kinesthesia, although not explicitly, aim to capture how afferent information is used by the internal brain processes to regulate motor control and coordination. This could be due in part to the fact that it is generally accepted that proprioception in the absence of muscle contraction (passive proprioception) is dependent only on the processing of peripheral inputs (Craggs et al., 1979; Nakajima et al., 2006). Indeed, the relative contribution of well-recognized processes to proprioception when the agent is in action, with muscle contraction with afferent and referent signals (active proprioception), remains unclear (Proske and Gandevia, 2012).

A closer look reveals the striking difficulty that we address in this section: the role of afferent information within the context of movement control and coordination. Theorists supporting internal models for motor control have expressed a clear position in this debate^3^. This is based on a recognition of proprioception as the means to provide the agent with a variety of crucial information for motor learning to occur.

These theories have been used to understand how the agent perceives the difference between self-initiated voluntary own actions (sensory reafference) and passive, involuntary, and unexpected (so-called sensory exafference) movements (Proske and Gandevia, 2012). Voluntary and accurate motor performance depends on self-generated reflexes, from nervous pathways to each muscle via spinal interneurons, and on a predictive CNS internal model to overcome noise in proprioceptive receptor signaling (Wolpert et al., 1995; Wolpert and Ghahramani, 2000). In turn, this anticipatory signal is subtracted from the incoming sensory signal to cancel the self-generated portion (a reafference), and create a neural representation of the outside world (an exafference) (Crapse and Sommer, 2008). Learning occurs as a result of the continued interaction of proprioceptive feedback and motor performance, thus, strengthening the reference mechanism and allowing the newly acquired skill to become part of the agent's repertoire of learned movements. Once a motor skill becomes automatic, its performance is under the control of a motor program. More recent research has generalized this idea by sustaining that an internal prediction of the sensory consequences of our actions—a copy of the motor commands to muscles as a centrally represented movement pattern stored in memory—is compared with actual sensory afference (Mitsuo et al., 2003; Wolpert et al., 2011).

In short, neural control centers are thought to predict and specify the motor commands required for active (self-initiated) movement (Farrer et al., 2003; Capaday et al., 2013). These rich internal models work similarly to a B-format; they “represent states of the subject's own body and, indeed, represent them from an internal perspective” (Goldman, 2012, p. 73). Briefly, they are doing all the functional work of proprioception regardless of the role of the body and its relationships.

We, however, believe that this may be problematic. The motor command specifies a precise value for a parameter of position, speed, or other, a corresponding unique value at the level of the sensor, with the variations being totally determined (Piaget, 1937; Lenay, 2006). As Gapenne (2014) asserts, this hypothesis emphasizes the existence of a bijective relation between action and sensation in the case of proprioception that “primes the subsequent inferences realized by the ‘brain,’ [which] are produced ‘at random’ remains mysterious […] Where do these commands come from? Why do they take the form that they do? Are they generated by a ‘program’?” (Gapenne, 2014).

In contrast to this position, we could think that active proprioception—in a PK system—is something the agent does in a particular situation and in an ongoing fashion. For example, it is certainly relevant in the motor control for an active human agent to walk on a swaying tightrope or for a spider caught on her windblown spiderweb. Both must fine-tune their muscle activity to maintain posture, coordinate sequential movements involving multiple joints, or be prepared for the next move and to stay upright. This motor command would be more than just a matter of pure effectuation that depends on an updated internal representation of body position during the production of learned movement. In this case, the agent would not be able to have access to any variations other than those produced by their own actions—an idea that denies the importance of the various forms of activity of the sensor interacting with a dynamical environment.

From this point of view, phenomena such as gravitation or friction always leave a certain degree of uncertainty concerning the movement which will actually occur (Henri, 1902). These variations, as Gapenne (2014) claims, which cannot be determined by the command, are actually a condition for the possibility of constituting an experience of the spatiality and temporality of the body/self in the present time, or toward accurate coordination with the environment on the basis of the constant and actual variations. This is true even when, as we have already stated, this PK perceptual experience involves the full set of sensory organs.

There is some evidence in support of an interpretation of PK-coupling in sensorimotor theory terms. For instance, a study in which subjects were asked to apposite the index fingertip of one hand to that of the other hand, found that the index fingertip was localized with equal accuracy and with no greater variability when the hand was moved actively by the subject or passively by an experimenter (Darling et al., 2018). The study found the differential activity of the sensor when interacting and no evidence that accurate proprioceptive localization or motor performance depended on the predictions of a CNS internal model to overcome noise in proprioceptive receptor signaling (Darling et al., 2018).

Consistent with this finding, studies conducted in light of the theory of referent control of action and perception (Asatryan and Feldman, 1965; Feldman, 2016), propose that to produce intentional motor actions, the nervous system changes specific neurophysiological parameters—the spatial thresholds at which muscles begin to be activated. When changed, these parameters shift the equilibrium state in the interaction between the organism and the environment^4^. Therefore, these parameters do not result only from the meaningful perception of the B-format, but also from the perception of proprioceptive-kinesthetic coupling with the body situated in the actual environment, with dynamic possibilities for action and oriented with respect to the direction of gravity. As Feldman (2016) proposes, the emergence of optimal sensorimotor action happens without preprogramming due to the cooperative tendency of neuromuscular elements to reach the shifted equilibrium state.

Based on this type of evidence, and moving forward to internal model descriptions, we argue that proprioception goes beyond a positional sense and the preprogramming of motor commands. The PK system would be the origin of spatial frames of reference in which neuromuscular elements are commanded to work (Feldman, 2016). Moreover, in the distinction between active and passive movement, we assert that the agent, with her own activity, is sensitive to the effects of her own actions and to the variations of the afferent signals. This moto-proprioceptive coupling allows the emergence of a continuous and dynamic reference to calibrate other sensorial signals through action (Iscla and Blount, 2012; Lebois et al., 2012). Accordingly, Gapenne (2014) supports that the singularity of proprioception lies in the fact that it is a firm reference-point, a mechanism of “filtering and calibration,” which allows an agent to dissociate between self and world, by attributing variations either to her own activity (and thus to the effects of her actions) or to events over which she has no control (Henri, 1902; Gapenne, 2010).

ESMT provides us with a more coherent account of these conceptual issues and findings, taking into consideration agents acting in everyday life, crossing their arms or walking fast to get to work, or avoiding losing their balance when the subway makes a sudden stop. The agent continuously tries to adapt to the disturbances and to recognize meaningful interactions. Noticing this PK coupling nature in perceptual experience and developing a framework unconstrained by the limitations of the current accounts, will be the goal of the rest of the paper. In the following sections, we propose how a description based on ESMT, with deeper links to phenomenology, can contribute to a better understanding of the PK perceptual experience in active agents.

## Overcoming the Bias: Three Kinds of Proprioceptive-Kinesthetic Contingencies (PK-SMC)

In a similar way to the ecological approach, in the enactive approach to cognitive science “perception does not consist of the recovery of a pre-given world but exists rather in the perceptual guidance of action in a world that is inseparable from our sensorimotor capacities” (Varela et al., 1991, p. 17). This view rejects mainstream theories of perception, which claim that perceiving is about giving rise to internal mental representations from the external world. In this respect, Varela et al. (1991) realizes that a foundational concern in developing this theory, which replaces representations with embodied action, is “to determine the common principles or lawful linkages between sensory and motor systems” (Varela et al., 1991, p. 173). Indeed, cognition is understood as a hands-on practical activity taking place in concrete situations (Varela et al., 1991).

ESMT, as a philosophical and scientific research program (e.g., O'Regan and Noë, 2001; Noë, 2004; O'Regan, 2011), has been developed with a similar concern^5^. Accordingly, perceiving is a bodily skill exercising an implicit know-how of the systematic ways that sensations change as a result of potential movements, that is, of sensorimotor contingencies (SMCs) (O'Regan and Noë, 2001; Silverman, 2018). Thus, perceptual modalities differ because they relate to a particular set of exploratory bodily movements: visuo-motor, auditory-motor, proprioceptive-kinesthetic, etc., which together constitute a detailed, directed, and unmediated awareness and allow access to the environment. Stemming clearly from a background of ecological laws, the properties of the SMCs related to the environment are the most general kind of regularities or so-called “laws” of SMCs.

In the following, we suggest that a felt PK perceptual experience is inseparable from sensorimotor expectations. We describe these PK-contingencies as depending on the awareness of the self's potential actions and interactions, abilities that an agent may acquire over a particular history of learning within a specific ecological and self-other environment.

###  Phenomenology and PK-SMCs

As a means of distinction, ESMT is not only an account of the lawful linkages between sensory and motor systems involved in perception; it also has set itself the much more challenging task of explaining the felt aspect of phenomenal consciousness. It assumes that experience is not caused only by some internal correlate, such as a B representation; in the words of Myin and O'Regan (2002, p. 33): “phenomenality is not caused by some brain process, but is constituted by the different capacities that ‘feeling’ involves.”

But what is special about the proprioceptive and kinesthetic conscious experience that makes it different from other mental phenomena, such as inference thought or color perception? To some extent, when framing the phenomenology of bodily awareness, we can consider the difference between not paying specific attention to our body and actually feeling an exasperating itch in the right leg. In this respect, proprioceptive awareness has been found in philosophical literature related to three domains of experience: the sensation of body position or the sensation of the location at which I feel my hand making the sign of peace occurring (sensorial information from specialized mechanoreceptors); first-person experiences of the sense of body ownership (the awareness of the hand that making the sign as being my own); and ecological self-experience, which is described as the ability to converge many relational aspects into a coherent identity (De Vignemont, 2018).

In particular, in this section, we are motivated by the domain of experience about what is it like to feel one's limbs along with their possibilities for action as one's own? So, we make a critical remark on the view that the felt location of bodily sensations suffices for the sense of bodily ownership (Crane et al., 1992); we favor the possibility that the phenomenology of ownership is over and above bodily sensations and that it is rather a feeling of bodily presence, as De Vignemont (2018, p. 44) proposes: “For instance, when something brushes our knee, not only do we feel a tactile sensation, we also become suddenly aware of the presence of our knee as being located in egocentric space, as a body part that we can reach and grasp. The existence of such a feeling is well-illustrated by amputees who still feel as if their lost limb were still there, physically present.”

This proposal is close to holding an action-based theory of perception, as an ESMT view of perceptual awareness. Indeed, the notion of the feeling of presence has originally been proposed from ESMT to characterize the detailed visual phenomenology associated with actual integrated scenes, even though the depicted scenes are not co-present at once (Noë, 2004). Feeling a body as present involves being aware of it as a whole object located in space and time, such as a sponge that one can explore from different perspectives and that one can actively manipulate. It is true that ESMT is particularly compelling for the visual and auditory modalities; however, the inherently exploratory nature of PK perceptual experience helps to account for the fact that PK perceptual experiences have a special phenomenal quality, that is not shared by other mental phenomena; and we can clearly see how perception-action coupling enriches the perceptual experience.

Thinking in PK perceptual experience as a feeling of bodily presence may provide powerful reasons for thinking that PK perceptual experience is constituted as the exercise of an exploratory bodily skill, which is refined as a result of expertise. Whenever the agent is effecting an actual change by self-movement, it has the effect of improving the veracity of attentive and sensible perceptual experience by confirming the anticipated sensorimotor regularities. Furthermore, if the PK conscious experience is constituted by potential exploratory movements it may turn out to be misleading, which has been amply demonstrated in the case of the bodily illusions when being wrong about own body's sensations and body awareness, such as in the Pinocchio illusion (Lackner, 1988) and rubber hand illusion (Botvinick and Cohen, 1998).

This solid connection between perceptual experience content and possibility for action is not new; it is crucial in Merleau-Ponty's “Phenomenology of perception” (Merleau-Ponty, 1945), in Gibson's affordance conceptualization (Gibson, 1977), and in Dreyfus's description of perception as a skill (Dreyfus, 1996). Here is where skill theories provide a route to naturalizing phenomenology: in this view, perceptual experience is not caused only by internal models but consists of various abilities that organisms have to feel, sense, move, grasp, respire, and interact. In order to explain the experience, therefore, instead of searching for neural correlates that ingrain phenomenality into electro-chemical mechanisms within the central nervous system, it is necessary to describe each of the different abilities that the organism displays when it engages in the perceptual activity.

Perceptual experience is shaped by that ongoing interaction with an environment at a present time, where manifold sensorimotor contingencies are at play. However, clearly not all of that SMCs are accessible to the organism's perceptual awareness at the conscious moment of “now”,—Varela (1999) shows that this moment has a duration of 1–3 s. indeed, some of these are realized by associated exploratory movements, and others are left out. As Myin (2016) argues, an organism has acquired, on the basis of a history of interactions, a sensitivity in its perception and action for each interactive generality that consists of implicit know-how.

However, it is not yet entirely clear what this phenomenal basis of PK perception means for the agent's experience. There are at least two possibilities, which we will refer to as perceptual sensitivity and perceptual awareness following Noë and O'Regan (2000) and Noë (2002)'s general distinctions, respectively:

1. PK-perceptual sensitivity: In general, this possibility comes from the habitual perceptual coupling of an organism and environment that lies in the history of previous interactions, that is, in the organism's coupling history with its physical and social world. O'Regan and Noe (2002) identify the sensation with a pattern of skillful activity. In ESMT terms, this means the perceptual experience of mastering sensorimotor contingencies (Froese and González-Grandón, 2019). When referring specifically to PK as a way of doing things, this sensed experience is a basic perceptual sensitivity of knowing how it feels to move the body even if the agent cannot directly sense all their body segments or lengths of joints simultaneously. Following Husserl's “habitual consciousness” conceptualization, Sheets-Johnstone (2019) describes this kind of sensitivity as an ongoing presence constituted by mindful bodies sensing themselves and their habitual relationship to the world.
2. PK-perceptual awareness: This possibility focuses on what the coupling affords, to be aware of each detail, and, although it is the result of the mastery of the relevant SMCs (Noë, 2002). It also consists of being aware of our immediate perceptual access (O'Regan and Noe, 2002). A feeling experience has qualitative dynamics of some individual kind, such as abrupt, slow, unexpected, or contractive, or combined when action or interaction unfolds. Living humans are not consciously aware of everything that their bodies do. But sometimes, when being alerted by something significant, such as a sudden cramp or tremor in one leg, this particular felt quality invites us to choose a particular pattern from among others, allowing it to play a prominent role in the embodied organism's present occurring actions (Myin and O'Regan, 2002; Myin, 2016).

PK-perceptual sensitivity as a possibility implies that specific ways of perceiving involve specific movements. When a person bends over to button up their shoelaces, for instance, she is not aware of each of her precise movements or postures through the ongoing activities. In describing this distinction, Noë (2002, p. 569) makes the following interesting observation: the driver, for example, who fails to pay attention to what he or she is doing or to a that to which he or she is responding to is still able to exercise mastery of the sensorimotor contingencies needed to drive the car. Such a driver is, as it were, on “automatic pilot.”

However, the possibility of PK-perceptual awareness is a matter of it being able to deploy a potential skill, namely integrating one's perceptual skills into one's intentional and spatio-temporal present action. This would imply that the agent is currently attending a sensorimotor contingency that has been previously learned. Moreover, following this distinction, the traditionally “intentional access” is not described in subpersonal terms anymore, as is the case with weak approaches. We may think about the possibility of accepting qualitatively different accounts: there must be some corporal mechanisms that are responsive to proprioceptive information from the entire body all at once, but others that differentially select between bodily parts. Then, as Fridland (2011) affirms, it seems that the PK conscious experience would be of multiple objects and would depend on the history, interests or plans of an agent. Although it would be rare to imagine proprioceptively and kinetically attending to the entire body in all its detail at once, following these ideas, it could be achieved with training.

Being more specific, coming from ESMT, PK knowledge-how may be identified with bodily skill rather than with possessing a B-format representation. Following the proposed distinctions, skilled PK-perceptual experience can be understood in terms of two key characteristics of PK-interaction, one habitual and the other more attentive, both presenting some kind of continuity, which is evident in perceptual learning. That is, PK perceptual experience is claimed to be constituted by the bodily skill of knowing how proprioceptive/kinesthetic sensations would change as a result of potential overt body movements. This is where implicit know-how constitutes this experience in terms of the perceptual accessibility of the currently non-accessed detail, and explicit know-how constitutes the highly attentive experience that assesses which potential PK-SMCs we should become aware of.

###  PK-Phenomenal Experience and Some Pieces of Evidence

Given the issues raised above, if PK awareness is to qualify as a legitimate form of awareness and not just subpersonal information, we can follow O'Shaughnessy (1995) and Fridland (2011) when arguing against having two separate explanations for conscious and subpersonal proprioceptive processing. From a phenomenological and ESMT stance, PK perception is not only about whether there is “something it is like” to experience parts of the body as own, such as a “sense of body ownership”^6^ but an immediate and direct first-hand or first-body experience with a felt qualitative dynamics.

Husserl (1989) describes the kinesthetic experience in terms of its qualitative nature: the dynamics of movement. In this sense, Sheets-Johnstone (2020) may reinforce the position in which it is not just a pre-reflective awareness of own body that is not very detailed, as proposed by Gallagher and Zahavi (2012, p. 155): “these postural and positional senses of where and how the body tends to remain in the background of my awareness; they are tacit, recessive. They are what phenomenologists call a ‘pre-reflective sense of myself as embodied’.” Instead, consider Sheets-Johnstone's description: “When we move, we kinesthetically feel the dynamics of the movement as they unfold, and insuppressible qualitative dynamics. A specific sensuous quality is indeed kinesthetically experienced” (Sheets-Johnstone, 2020).

In fact, following Husserl and her position, the description of the PK perceptual experience becomes more robust as it comes along with a sense of body posture and movement relative to the interaction with the environment. The agent feels a PK sense of her own body parts and their potential movement in relation to something or someone. In this regard, this view is much closer to the notion of “ecological self” from Neisser (1988) when describing this PK sense of dynamical self as an interactive body to produce sensations about the own movements in the ongoing interaction.

Consider the following basic example: when crossing your arms it is not simply necessary to register where your arms are positioned in space for the sake of knowing where your arms are as if you were solving a problem. Rather, this is a directly perceived and pragmatic problem: if you want to give someone a hug, you have to know what position your arms are in, how far or close the person you want to hug is, how much friction you have in terms of the clothes you are wearing, if the ground you are standing on is tilted, etc. This does not involve a theoretical reflection but a characteristic PK perceptual know-how: your bodily action is ready to go. PK accounts for one's ability to detect limb position and bodily posture from the inside, and it consequently has to be in a constant relationship with ecological interaction.

In a nutshell, this strong embodiment thesis helps us to describe in greater depth what PK-coupling feels like; it considers that this experience is about a spatio-temporal presence and is foundationally grounded in the skilled kinesthetic body (Sheets-Johnstone, 2020)^7^.

We can already note that these theoretical possibilities, in the framework of PK on the neurophysiology of motor behavior, attest to the importance of body awareness in proprioceptive perceptual learning. Feldman (2016), when referring to self-initiated movements at which muscles begin to be activated, rather than giving an absolute role to the afferent feedback, suggests that the central influences on the neuromuscular periphery (motoneurons) have an interactional and dynamic dimension.

There is also evidence, considering the unloading reflex—the reflex inhibition of the muscles of mastication that occurs when food or other material between the jaws suddenly collapses and helps to stop the jaws forcefully coming together—as an example of involuntary action. Ilmane et al. (2013) demonstrated that the corticospinal and other descending systems maintain the referent position of the wrist during unloading, thus, allowing the neuromuscular periphery (in the continuous and dynamic organization with central influences) to change motor commands and the wrist position in response to unloading, as an external and surprising perturbation.

Another source of evidence that is consistent with these findings comes from the kinesthetic illusions elicited by the tonic vibration of the tendon of an elbow flexor (Eklund, 1972; Goodwin et al., 1972). Vibration enhances the activity of flexor spindle afferents, eliciting an illusion of elbow extension as if elbow flexors were stretched. Most interpretations of this illusion argue that it results from an increase in the afferent component, while the central component remains unaffected by vibration. Here, again highlighting the importance of the whole percepto-motor system, Feldman (2016) suggests that the illusion can be explained by the influence of vibration on the central component, resulting in an actual motion-learned and reliable (meta)stable pattern in the sensorimotor coordination (Buhrmann et al., 2013).

Thus, to account for the constitution of this particular felt bodily experience—the immediately felt qualities of the experience of spatial and temporal self-orientation in action, such as in feeling oneself being the one acting, for example—the agent must learn to qualitatively distinguish between three sources of variation in the PK sensory signals that become coupled within an open-loop fashion in the online interaction: PK-SMCs self, PK-SMCs self-ecological, and PK-SMCs self-other.

In the following section we introduce and describe each of these PK-SMCs, analyzing the main conceptual points related to ESMT and kinesthetic phenomenology, and we also offer a formal description of each of them that leads to the development of our PK minimal model.

###  Proprioceptive-Kinesthetic Sensorimotor Contingencies-Self: PK-SMCs-Self

A key characteristic of a PK system is its sensibility or awareness of its own musculoskeletal parts in relation to other parts of one's body and of their possibilities for action and interaction. The PK-SMCs-self contingencies are described in this regard as involving the exercise of a bodily skill, the know-how of the systematic ways that a sense of the bodily self changes as a result of the potential moving self, in relation to one's body. We propose that all the aspects of the phenomenology of the sense of proprioceptive and kinesthetic coupling are related to both this inherent self-oriented sense in space and in the present time, and also in relation to perception and action cycles in interactions that together comprise the PK-SMCs-self kit. For instance, the experience of sensing the positions of body segments and their possibilities for movement in relation to each other. Certainly, the relational features always involve the physical and social world in the first place, and they do not require internal comparison between B-formats; in this section, however, we will only focus on the contingencies of the spatial and temporal orientation of the body's own parts and its possibilities for action, leaving for the following sections the establishment of meaningful relations between ourselves and the ecological and social world.

Moreover, in addition to the afferent signals of limb position that provide the central nervous system (CNS) with information about the spatial orientation of the body's own parts, the PK-SMCs-self also involves efferent signals, environmentally sensed constraints, such as gravity and friction, or the sensation of movement of another agent. The PK-SMCs-self are thus constitutive of the sensorimotor exploratory behavior of any human agent, as a form of baseline behavior to the ecological self, and are also enablers of self-other interaction.

The importance of the PK-SMCs-self as felt is also evident in the case of deafferented agents who lack PK perceptual awareness in a large part of their body. Although rare, some viral infections can cause autoimmune reactions that selectively attack the peripheral nervous system and destroy afferent pathways that are part of the PK system (Connell et al., 2008). In these cases, subjects no longer have proprioceptive awareness in the parts of their bodies affected by neuropathy. They lose the ability to immediately recognize their practical possibilities for action. But since this condition does not affect the efferent nerves, and it is still possible for subjects to regain the ability to produce movement with those parts that they can no longer feel but can visually perceive. This had been taken to show that proprioceptive awareness is not necessary for bodily action (Bermúdez, 2000; O'shaughnessy, 2008).

However, we argue that in the absence of the PK-SMCs-self set, ordinary action as we know it is impossible. Deafferented agents have severe problems in the online control of action, and their actions may seem performed distant because of lacking PK perceptual sensitivity and awareness. When a deafferented agent does not sense or feel their limbs and uses her attentive gaze instead, she loses the possibility of experiencing her orientation in relation to the limits of her own body and directly perceiving the possibilities for acting and interacting with her surroundings (Howe, 2018). Certainly, a deafferented subject with a lot of training will be able to achieve better possibilities for acting and interacting, and a form of awareness may arise, but it is not a PK perceptual awareness.

We argue that to recognize the difference between a skillful PK perception, from one that is not, or between the sensitive or aware qualitative dynamics variety, between habitual experience from paying attention to one's muscles movement and interaction possibilities, is a challenge that can be better understood regarding skilled PK-SMCs-self, where one of the two following possibilities must be at play:

– Skilled PK-SMCs-self (SPK): this possibility comes from taking into account the mastering of PK-SMCs-self. A PK-SMCs-self skilled agent has a learned perceptual sensibility, a widely recognized repertoire of body orientation, and concrete action possibilities in particular contexts from which a specific contingency can be selected for attention. This skilled agent therefore also has a PK perceptual awareness.
– Non-skilled PK-SMCs-self (NSPK): In contrast, this possibility comes from considering agents such as those who are deafferented or live with some similar affectation. The PK-SMCs-self have not been developed properly, and the agent thus does not recognize the limits of their own body and the possibilities for acting and interacting with their surroundings in a practical way. As a deafferented PK agent whose perceptual experience is disconnected from their practical possibilities.

One way of shaping these intuitions is to formalize the PK-SMCs-self of an agent with the environment through a dynamic systems approach. There have been a few attempts to define SMCs on a strictly formal basis, although with less emphasis on proprioception. Philipona et al. (2003), for example, trying to deduce the dimensionality of the external space of interaction of an agent, proposed an algorithm to capture the position based on inputs and outputs.

For our purposes, inspired by the work of Buhrmann et al. (2013), we chose some variables to describe the PK coupling, and we made use of a minimal dynamical model to describe the different kinds of sources of variation, the PK-SMCs.

###  PK-SMC-Self/Model Description

Inspired on the basic model for collective movement proposed by Vicsek et al. (1995), we considered the simplest case of only one agent moving continuously inside a 2*d* square region of length *L* with periodic boundaries. The agent has developed PK-SMCs, denoted by *p*. The model assumes that the agent has a constant PK perceptual skill during the dynamics, and *p* thus does not depend on time.

In general, such a system could be described by the next set of equations regarding the agent's position **x** updates according to the following:

(1) $$\begin{array}{l} \text{x}\left(t+1\right)=\left[\text{x}\left(t\right)+\frac{\xi_{1}\left(t\right)}{p}\right]+\kappa \theta \left(t+1\right) \end{array}$$

The first part of the right-hand side of the above equation shows that the agent, in order to move, must perceive its position in the world. This perception is portrayed by the whole first big parentheses of Equation (1), and it is influenced by three things: the real agent's position **x**(*t*), the agent's PK ability *p*, and other factors that are not explicitly described in the equation but are implicit in the variable ξ_1_(*t*). These could include both external stimuli and internal mechanisms that do not depend on the PK ability but could modify the agent's perception. Going back to the example of the earring, this variable ξ_1_(*t*) could be an unexpected disturbance such as an involuntary handshake or a shove from another person that could alter the agent's perception of their orientation and could have an impact on the final task of putting the earring into. This variable ξ_1_(*t*) is a random variable taken uniformly in [−ξ, ξ]^8^^,^^9^^,^^10^. Then, if the parameter ξ > 0 is low, the perception of the agent depends mostly on its PK ability: if the agent has a good PK ability (high *p*), their perception of their position would be very accurate, but if they have a poor PK ability (low *p*), her perception would be wrong; if ξ takes medium values, then the agent's PK ability, if good, could absorb its effect. But if the agent's PK ability is bad, then ξ could amplify an already bad perception; if ξ is high enough, it does not matter if the agent has a good or bad PK ability, as the effect of ξ will cause its perception to be wrong. Below we will specify what we mean exactly by “small,” “medium,” and “high enough.”

The second part of the right side of Equation (1) updates the agent's direction and, consequently, updates its position. It portrays the fact that the agent also needs to move in order to perceive, as was proposed by Gibson (1977). The agent's direction is given by θ; an angle between −π and π, and is defined as:

(2) $$\begin{array}{l} \theta \left(t+1\right)=\theta \left(t\right)+\frac{\xi_{2}\left(t\right)}{p} \end{array}$$

In order to sum this angle to the agent's positions, it is transformed in a 2*d* vector defined as [cos(θ), sin(θ)]. The random variable ξ_2_(*t*) is interpreted as before: a random variable taken uniformly within the interval [−ξ, ξ]^11^. A Skilled PK-SMCs-self (SPK) then implies that the agent is more aware of their possibilities for movement, and a Non-Skilled PK-SMCs-self (NSPK) implies the opposite. For simplicity, we assume that the length step between updates is given by the factor κ. This ensures that the agent's movement is at a constant velocity in direction of θ.

The minimal model thus incorporates our previous proposal that proprioception is coupled with kinesthesia: the agent senses its body and performs it. Based on this, we predicted that an agent with SPK will be better aware of this own position in space and movement possibilities; as a consequence, its future movement will be less erratic than an agent with NSPK.

**Figure 1:**
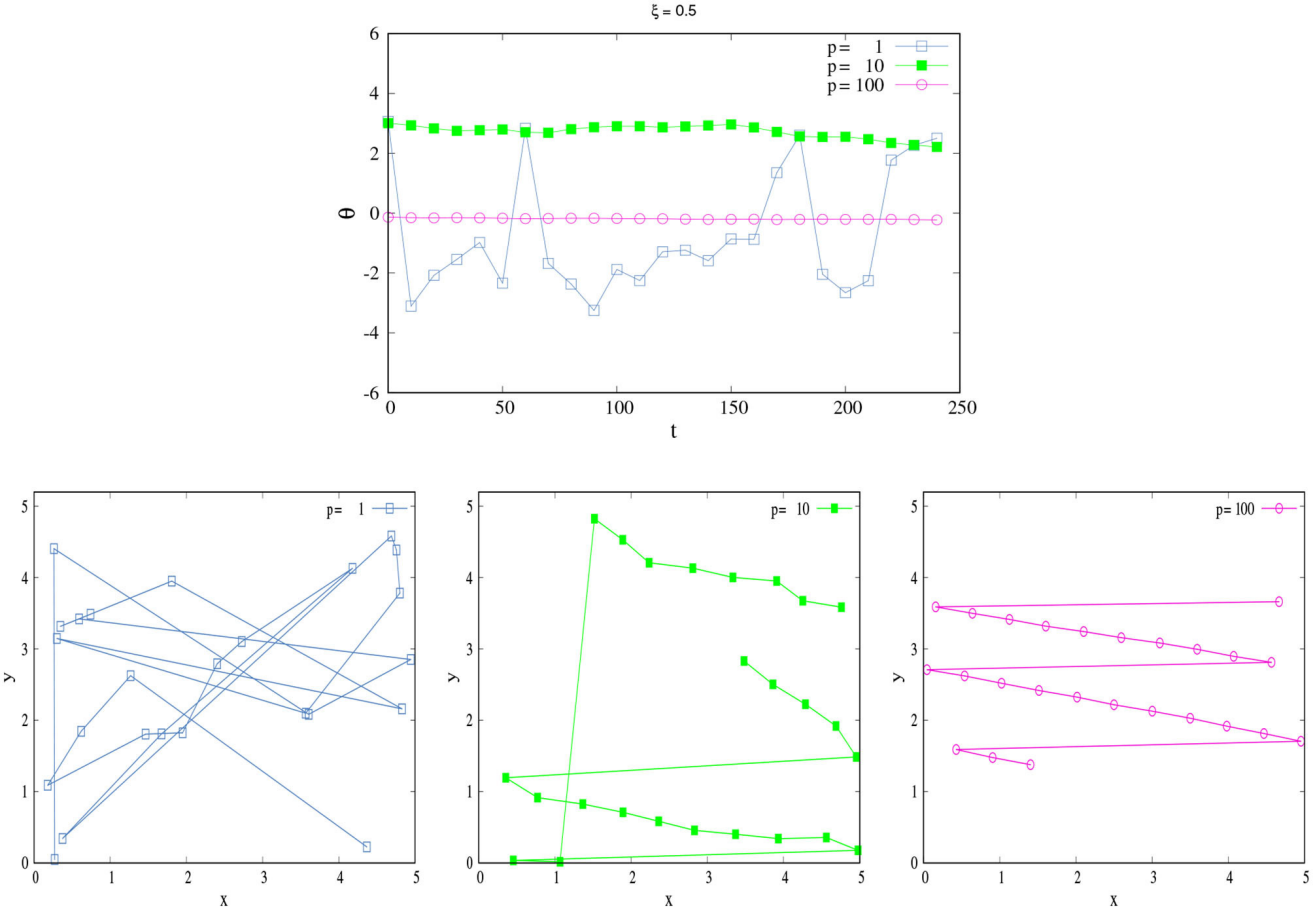
SPK vs NSPK. **(Top)** Change of θ in time for different values of *p*. **(Bottom left)** Trajectory of an agent with low *p* = 1. **(Bottom center)** Trajectory of an agent with medium *p* = 10. **(Bottom right)** Trajectory of an agent with high *p* = 100. Each trajectory (different color) corresponds to its respective color curve in the changes of θ. Here, ξ = 0.5 and κ = 0.05. All these curves correspond to only one simulation for a single agent in a 2*d* square space of length *L* = 5 with periodic boundaries. Here, *t* = 250, and the curves show every 10 time steps for a better visualization. The large jumps are due to the fact that the space has periodic boundaries; when one coordinate (*x* or *y*) in the agent's positions is too close to the boundary, it appears in the other side of the space.

In order to illustrate the last affirmation, Figure 1 compares the trajectories in the space of a SPK agent and NSPK agent. As we explain above, the agent's movement will depend on the parameters ξ and *p*—the combination of which will give us different behaviors. In order to study the effect of each one we first fixed ξ = 0.5 and observed how **x** and θ changed in time for different values of *p*. The agent moves in a 2*d* square of length *L* = 5 with periodic boundaries and κ = 0.05, i.e., it travels 0.05 units in each time step. The total time of the dynamics is *t* = 250. The initial angles and positions to start the dynamics were taken randomly.

Figure 1, top displays the change of θ for different values of *p*, and we can see that if *p* is small (=1, blue squares) the agent shows very drastic changes in terms of their angle movements due to the large effects of the external perturbations [ξ_2_(*t*)], implying that the agent does not have the skill to act in harmony with their world. This lack of SPK also influences the agent's spatio-temporal self-orientation; she consequently travels erratically in the space because she does not know her exact position in the world, displaying an erratic trajectory with changes in position and direction (Figure 1, bottom Left). This behavior changes as *p* grows: when *p* = 10 (green filled squares), the changes in θ are not so drastic and the trajectory now shows smaller fluctuations. With these values of ξ and *p*, the agent is more aware of their spatial position and possibilities for movement, making a somewhat more organized trajectory (Figure 1, bottom Center). When *p* = 100 (pink circles), the agent is fully SPK as a result of an active coupling between performing and sensing. The fluctuations in θ are practically nonexistent, and its trajectory is fully organized (Figure 1, bottom Right)^12^.

**Figure 2:**
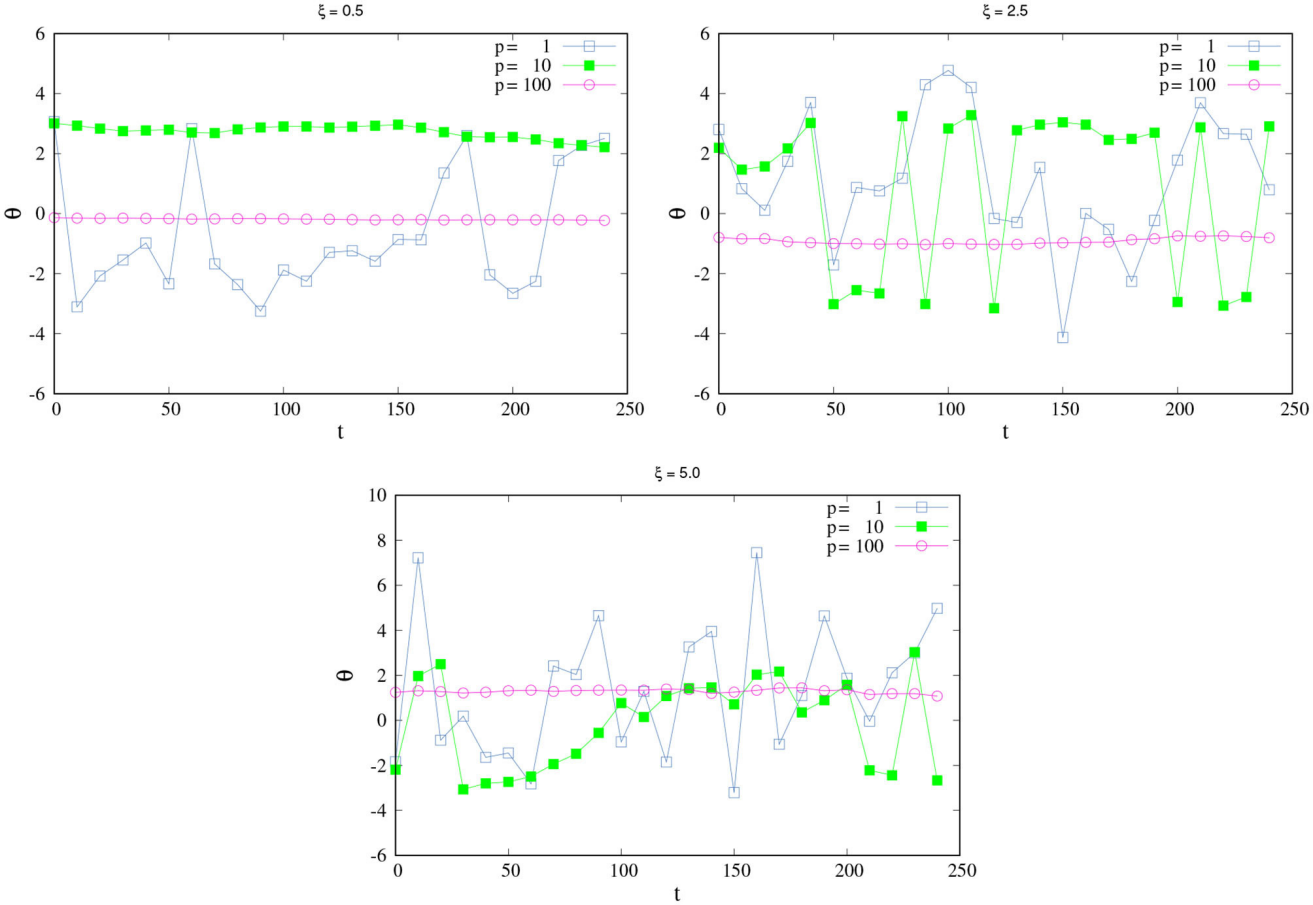
How SPK and NSPK change when noise increases. Change of θ in time for different values of ξ and *p*. **(Top left)** ξ = 0.5. **(Top right)** ξ = 2.5. **(Bottom)** ξ = 5.0. All remaining parameters take the same values as in Figure 1.

Figure 2 shows the change of θ as function of *t* for different values of ξ and *p*. When ξ is small (Figure 2, top Left), an agent with medium *p* is SPK, as we discussed above. When ξ increases, high values of *p* are necessary to reach the SPK. For example, Figure 2, top right shows the case ξ = 2.5, here an agent with *p* = 10 is not SPK anymore; the changes in its direction are too drastic, it would need a higher *p* to be a SPK agent. At values of *p* = 100, the agent can resist higher values of ξ; here, the agent is completely SPK and responds well to high values of noise. An analogous situation for this last scenario (of a completely SPK) would be one in which the agent can insert an earring while they are in a moving car on a very irregular pathway or even when their hand is wet and the earring is very tiny.

We can say that this super SPK agent not only has a great PK-perceptual awareness but also high PK-perceptual sensitivity. Her great response to noise and ability to nullify it not only comes from their high PK-perceptual awareness (integrating her purely perceptual skills into intentional and spatiotemporal present actions) but also from their PK-perceptual sensitivity, which gives them the ability to respond efficiently and automatically to high levels of noise that could otherwise affect their conscious actions. Then, the PK-awareness and the PK-sensitivity are correlated in the sense that a high PK-sensitivity gives the agent better PK-awareness and, therefore, a super or complete SPK.

From these results, we can say that an SPK agent is one whose parameter *p* is high enough to compensate for the effects of noise in the skilled exercise and awareness of the implicit know-how of the lawful ways that sensations change as a result of potential movements. This concept will be extended in further sections but whilst maintaining this general idea. The model is based on established theories of SMC in the sense that it follows some of the descriptions set out in previous sections, although we arbitrarily select parameter values depending on the focus of interest.

###  PK-SMC-Self-Ecological

Proprioception has been largely described either as a subconscious process, as mentioned previously in relation to B-formats, in that it does not typically require directed awareness or attention or even doubted regarding its perceptual nature (O'Shaughnessy, 1995; Sydney, 1996; Bermúdez, 2000). For us, since we are interested in thinking about proprioception coupled with kinesthesia, as a form of awareness or as a percepto-motor skill that can be developed throughout the life of the organism, we emphasize the interactive co-dependence between the PK-SMC-self with the ecological environment that shapes specific modes of coupling. In this line, understanding sensorimotor patterns in a perceptual PK experience becomes relevant for explaining PK awareness as a skill in interaction.

In relation to the distinction made in previous sections between perceptual sensitivity and perceptual awareness Noë and O'Regan (2000) and O'Regan et al. (2004) take this distinction further and propose two other concepts to try to relate these concepts to body sensitivity and body awareness, respectively: “grabbiness or alerting capacity” and “bodiliness or corporality.” Similar to the idea of salience in the context of affordance ecological theory, “grabbiness” is associated with the contextual attractiveness of something to a perceiver related to the presence of mastering of SMCs. It also has a complementary aspect, “bodiliness,” which refers to how much the perceiver's perceptual awareness will change when the perceiver moves. The greater these changes, the higher the degree of “bodiliness.” It is worth mentioning that O'Regan et al. (2005), explicitly state that proprioception does not have “grabbiness”:

“Proprioception is the neural input that signals mechanical displacements of the muscles and joints. Motor commands that give rise to movements necessarily produce proprioceptive input, and proprioception therefore has a high degree of corporality. On the other hand, proprioception has no alerting capacity: changes in body position do not peremptorily cause attentional resources to be diverted to them. We therefore expect that proprioception should not appear to have an experienced sensory quality. Indeed it is true that, though we generally know where our limbs are, this position sense does not have a sensory nature” (O'Regan et al., 2005, p. 60).

First, we consider that the PK system, as a perception-action coupling, does have a sensory nature: the way we position ourselves and move in the world has a particular experienced sensory quality. As Sheets-Johnstone (2019, p. 150) states, action directs attention toward the dynamics of movement that precisely constitute qualitative dynamics, “whether a matter of self-movement or the movement of human and nonhuman animals and of objects in the world.” Now, what O'Regan et al. (2005) identified here is certainly the positional component of the PK system, suppressing the felt or perceived dynamics in the interaction. Whether an infant mastering their PK-SMCs to be able to get into a crawling position on their hands and knees as a form of perceptual sensitivity or body grabbiness or an adult learning a new skill, such as paying attention to a new clinical skill in preparation for medical training, the mastering of PK-SMCs and the acquisition of new skills requires a proprioceptive/kinesthetically-attuned body—a dynamic body that feels^13^.

Second, we consider that O'Regan et al. (2005) have left open how are we to understand the relationship between an agent interacting with the environment in a particular scene, such as those where affordances are sensitive to sudden changes in muscular tone or position and activate attentional resources to be automatically directed to the location of change^14^. According to Gibson (1977, p. 140), specific muscles, kinesthetic habits, attentional processes and preparedness, as well as one's own action readiness remain activated throughout the interaction with a particular environment. It is true that it may be less peremptory than in the case of vision or hearing, but grabbiness is also present. Indeed, the claim of ESMT is that the orientation responses primed by the grabbiness of interaction constitute the qualitative feel of PK perceptual experience. In this respect, we argue that PK-SMCs self-ecological also possesses a high enough degree of body sensitivity and awareness with “grabbiness” and “bodiliness.”

Drawing on these distinctions, ESMT seems to provide a unique perspective on the consistent description of PK perceptual experience as constituted by a variety of bodily skills. We consider that among human agents, the strategies to be mastered or skilled are always at the interface with the ecological environment and its norms and the social environment.

Indeed, the development or acquisition of particular PK-SMCs describes how an agent becomes attuned to a specific ecological interaction by regulating, selecting (as it is preferable to act more optimally in the known environment), or modulating the relational patterns in accordance with relevant norms. PK-SMCs change as a result of learning and training. That is, it seems clear that proprioceptive awareness is dependent on what we know, how we act, and how we bring attention to our bodies. We refine our feeling of PK-SMCs, providing a pragmatic bodily awareness related primarily to the agent's posture, action possibilities and to constant action and interaction updating as a result of expertise (Gallagher, 2006, 2017; Tsakiris, 2015)^15^.

Although our model does not yet include variability in the forms of PK awareness in terms of parameters α and β as functions of *p*, in future steps of this research, we would like to better understand the qualitative dynamics diversity in the larger differentiation of this ability by including some of these variables in our minimal model.

###  PK-SMC-Self-Ecological/Model Description

**Figure 3:**
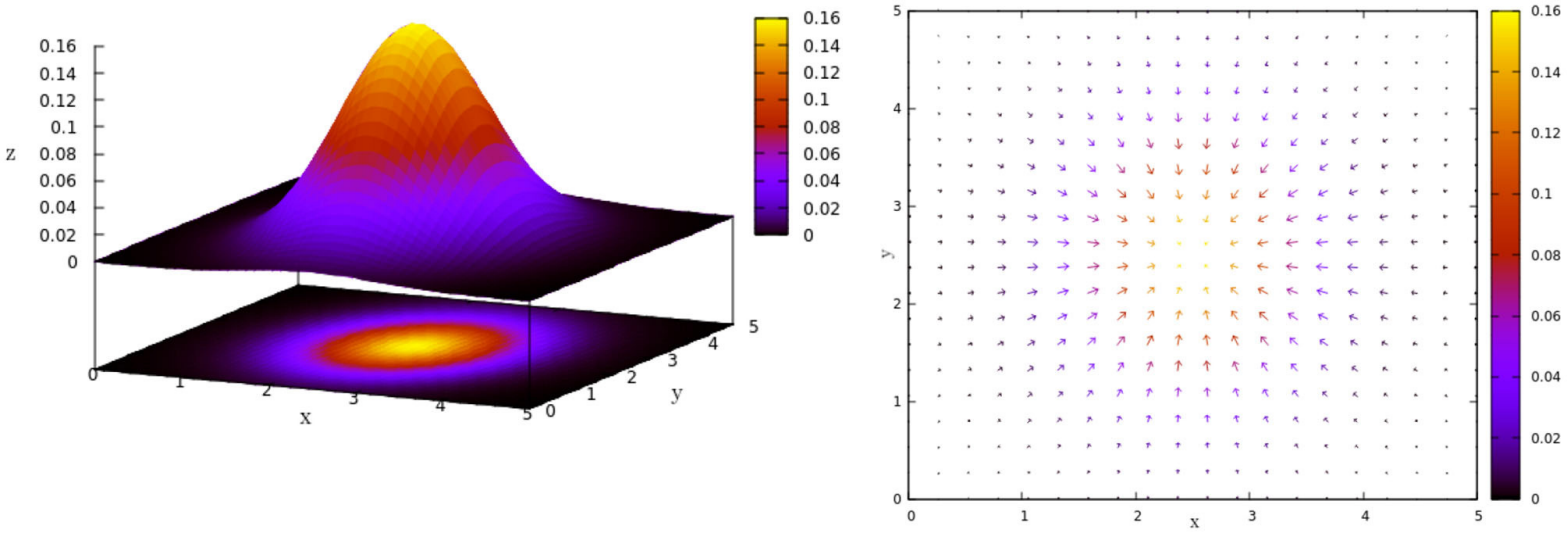
Heterogeneous space. **(Left)** Normal diffusion in [0, 5] × [0, 5] following Equation (3). **(Right)** Gradient vector field *G*, associated to Equation (3), which gives us the gradient vector **g** at each point of the space.

To include the interaction between an agent and the environment in our minimal proposed model, we will consider heterogeneity in space, a concentration gradient that diffuses in a normal way with origin in the center of the space of length *L*. This implies that for each point (*x, y*) in the space there is a concentration given by the following:

(3) $$\begin{array}{l} N\left(x,y\right)=\frac{1}{2\pi }\text{exp }\left(-\frac{{\left(x-L/2\right)}^{2}+{\left(y-L/2\right)}^{2}}{2}\right) \end{array}$$

as Figure 3, left shows for a space of length *L* = 5.

The agent will interact with this heterogeneous space through each gradient vector in the gradient vector field *G* given by $G:=\left\{\text{g}=\left(g_{x},g_{y}\right)=\left(\frac{\partial N}{\partial x},\frac{\partial N}{\partial y}\right)\forall \left(x,y\right)\in \left[0,L\right]\times \left[0,L\right]\right\}$ (Figure 3, right). Each gradient vector **g** describes in which direction and in what proportion the greatest change in the concentration occurs. To simplify the computations, we consider the normalization of **g**, i.e., **g** = **g**/‖**g**‖. The new agent's direction θ(*t* + 1) will be a weighted sum between the previous direction (θ(*t*)) and the direction given by the gradient vector **g** defined by the agent's actual position **x**(*t*). For this we must modify Equation (2) as follows:

(4) $$\begin{array}{l} \theta \left(t+1\right)=\alpha \left[\theta \left(t\right)+\frac{\xi_{2}\left(t\right)}{p}\right]+\beta \left[\theta_{\text{g}}+\frac{\xi_{3}\left(t\right)}{p}\right] \end{array}$$

with θ_**g**_ = arctan(*g*_*y*_/*g*_*x*_), ξ_3_ as a random variable taken uniformly in [−ξ, ξ], and α, β free parameters such that α+β = 1. Here, the noise variable ξ_3_(*t*) is interpreted as before: an skilled agent will be more aware of the effect of the environment in their movement, following it with more certainty and being able to interact with it effectively. The addition of new parameters α and β portrays the fact that the acting agent may make a distinction between two sources of variation in the sensory signals that affect it: one related to their own activity (α) and another related to their interaction with the environment (β). An SPK then allows the agent to follow (with a certain weight) the direction of the greatest concentration, i.e., the agent has a feeling of a specific type of coordination with opportunities afforded by the various degrees in which she interacts with their environment.

We want to investigate the effect of the PK value *p* on the interaction between an isolated agent and the environment (PK-SMC-self-ecological). We consider that an agent interacts successfully with their environment if it is capable of finding the origin of the concentration gradient. For this, we suppose that α = β = 0.5, i.e., the agent takes equally into account in terms of movement, their own direction, and the direction given by the gradient. We are going to consider the average success rate *s* and the average first-arrival time τ, i.e., how many experiments the agent was able to find the center of the concentration in and how long it took them to do so.

**Figure 4:**
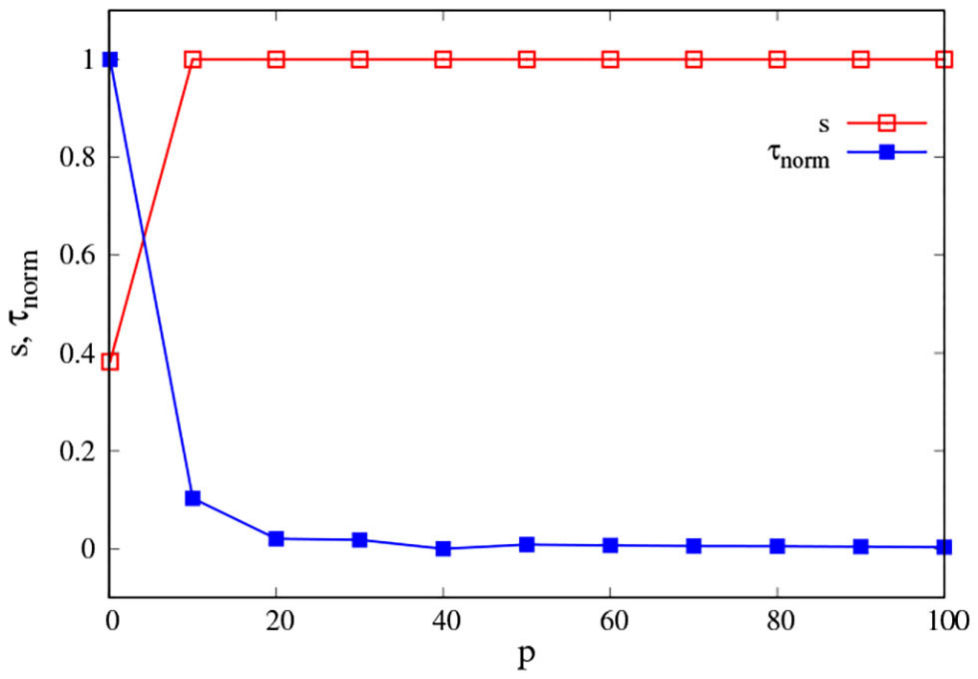
Environmental interaction. Average success rate *s* (red squares) and average first-arrival time τ (blue squares) as a function of *p*. The curves are the result of averaging 500 different experiments with *t* = 2, 500 each one, with ξ = 0.5 and κ = 0.05. For a better reading of the graph we considered τ_*norm*_ = (τ − τ_*min*_)/(τ_*max*_ − τ_*min*_).

Figure 4 shows the change of *s* and τ_*norm*_ as *p* increases. We see that for low values of PK *p* < 10 the success rate is low (red squares), and the average first-arrival is large (blue squares). This means that an NSA was not always able to find the concentration center; when they did, it took a long time. Their ability to interact with the environment was not good. On the contrary, if the agent has a PK value above 10 (SPK), they are capable of finding the origin of the concentration gradient at every time and also within a very short time in comparison with an non-skilled agent (NSPK). The effect of increasing the noise ξ is the same as before: an SPK agent could become an NSPK if ξ is high enough and their SPK is not sufficient to compensate for its effect in their spatio-temporal self-orientation in present action and interaction. We have explored the effect of α and β in more depth in the next model section.

###  PK-SMCs Self-Other: Can Sensorimotor Contingencies Account for Processes Such as Social Perception?

The aforementioned idea of ecological PK-SMCs can also be applied to the PK perception of another person. From enactive social cognition, it is known that the motor system is involved in social perception (Gallagher, 2009; Froese et al., 2020). More accurately, in line with ESMT, it has been suggested that social perception consists of the skillful co-regulation of participatory social interaction (De Jaegher et al., 2010). Each person needs to have knowledge of the qualitative dynamics caused by the other's bodily movements concerning their own possible bodily movements. The mastery of these “self-other contingencies,” as McGann and De Jaegher (2009) call it, provides a PK-self-other perceptual experience.

According to the strong position defended in this article, both social and ecological PK perception depends on skillful regulation of interaction with different invariants and qualitative dynamics. In each case, this includes perceiving the air as air or another person as another person. However, in this second form, intentional access or perceptual awareness additionally depend on a complementary skillful response by the other person. Both have to master PK-self-other contingencies. If the other agent does not respond appropriately, the PK perceptual experience would be more akin to that of ecological PK perception. Nevertheless, it is not yet entirely clear what this self-other basis of PK perceptual experience means for the agent's experience. There may be many instances for meaningful PK interaction, but we will concentrate mainly on two for the operational purposes of the description and the proposed model. We will refer to these as “PK-self-other sensitivity” and “PK-self-other awareness” forms of PK social perception, respectively:

1. PK-SMCs self-other sensitivity: In this case, one agent's perception of the other agent is only partly constituted by their ongoing social interaction, and each agent's perception can be molded by the other's movements possibilities but without constituting a meaningful shared moment of joint attentive experience. An example includes PK perceptual self-other sensitivity that may be evident in active daily interactions, which often require the agent to recognize the possibilities for the other to act and what their next move will be^16^.
2. PK-SMCs self-other awareness: This form gives rise to a jointly attentive unfolding experience because both agents have a mastering of PK-self-other contingencies. The more aware you are of those learned sensitive interactions, the more skilled you are in mastering self-other contingencies. In this case, there is a PK-SMCs-self other perceptual awareness in each agent to realize an attentive, skilled, and participatory performance. For instance, dancers of Argentinan tango can fluidly improvise together only when they actively explore their partner at every moment and reciprocally make their bodies amenable to being sensed (Kimmel, 2013)^17^.

What is important in this sensitivity and awareness context is to recognize not simply that during a human's history of coupling, others populate their self-dynamical space action possibilities or act as a reference point for the person's orientation in the present action, but that such interaction may also play a constitutive role in shaping human perception-action cycles and experiences. Indeed, an appropriate PK-self-other experience depends on adequate PK-SMC-self and PK-SMC-self-ecological. We propose that agents engaged in dyadic relations and particularly those having common PK-self-other awareness skills, are more easily able to include other agent's ecological self-action possibilities in their own ecological self.

We investigate these distinctions as a kind of minimal social interaction, arguing that PK self-other contingencies are constitutive of the varieties of PK-self-other experience, either in their sensitive or awareness qualities. That is, we assume that detecting the presence of others is a PK-SMCs-self-other that can be mastered and learned skillfully. Moreover, a skilled PK-self-other contingency is evident in activities like the above-mentioned dance or in sports that require interaction and trained interdependence to ensure a successful outcome. For example, the so-called alley-oop in basketball is an offensive play that requires both teammates involved to sufficiently know and feel the others' moves, one of them throwing the ball near the basket to the other teammate who jumps, catches the pass, and makes a basket (Doeden, 2014).

We advance in our minimal model proposal, based on the idea that an agent performing a jointly attentive unfolding experience directly incorporates ecological information relative to the agents in its ecological self-action possibilities, with PK-SMCs-self other awareness and sensorimotor learning.

###  PK-SMC-Self-Other/Model Description

The minimal PK model introduces social interaction considering two agents in space. Each agent *i* has its own PK value *p*_*i*_ and an interaction radius *r*. This interaction radius portrays the maximum reach of the agent's limbs. The position of agent *i* (**x**_*i*_) updates as Equation (1), and its angle θ_*i*_ is as follows:

(5) $$\begin{array}{l} \theta_{i}\left(t+1\right)=<\theta_{i}\left(t\right){>}_{r}+\frac{\xi_{2}\left(t\right)}{p_{i}} \end{array}$$

where < θ(*t*) >_*r*_ is the average angle inside of the interaction radius *r* of agent *i* (counting itself) and is given by < θ(*t*) >_*r*_ = arctan(< sin (θ(*t*)) >_*r*_ / < cos(θ(*t*)) >_*r*_).

The role of PK is interpreted in the same way as before: an SPK implies that the agent is more aware of their own orientation and their own activity when interacting with others. The agent has also developed PK-SMCs self-other awareness; an NSPK implies the contrary—that the agent has only developed PK- SMCs self-other sensitivity. The SPK agent will be also, and by consequence of its SPK ability, coordinating its movements with its partner when interacting.

In the case in which we consider the interaction between agents and the interaction of each one of them with the environment, θ_*i*_ is updated as follows:

(6) $$\begin{array}{l} \theta_{i}\left(t+1\right)=\alpha \;\left[<\theta_{i}\left(t\right){>}_{r}+\frac{\xi_{2}\left(t\right)}{p_{i}}\right]+\beta \;\left[\theta_{\text{g}}+\frac{\xi_{3}\left(t\right)}{p_{i}}\right] \end{array}$$

For the results shown below, we consider the simplest case in which only two agents move inside a square-shaped cell of linear size *L* with periodic boundary conditions. The agents are characterized by points moving continuously in the plane, and (as we discussed before) they have several capabilities:

- Each agent has an interaction radius r=1 centering in the agent's position **x**. So, if *d*(**x**_*i*_, **x**_*j*_) ≤ 1, the agents will interact between them, where *d*(**x**_*i*_, **x**_*j*_) is the euclidean distance between positions of agent *i* and agent *j*, with {*i, j*} = {1, 2}.
- Each agent *i* has the ability of PK denoted by *p*_*i*_. Here, we consider that *p* ∈ [0, 100].

Given these minimal assumptions, we remember that agents update their position as follows:

(7) $$\begin{array}{l} {\text{x}}_{i}\left(t+1\right)=\left[{\text{x}}_{i}\left(t\right)+\xi_{1}\left(t\right)/p_{i}\right]+\kappa \theta_{i}\left(t+1\right) \end{array}$$

with

$$\begin{array}{l} \theta_{i}\left(t+1\right)=<\theta_{i}\left(t\right){>}_{r}+\frac{\xi_{2}\left(t\right)}{p_{i}} \end{array}$$

in the case of PK-SMC-self-other, and

$$\begin{array}{l} \theta_{i}\left(t+1\right)=\alpha \;\left[<\theta_{i}\left(t\right){>}_{r}+\frac{\xi_{2}\left(t\right)}{p_{i}}\right]+\beta \;\left[\theta_{\text{g}}+\frac{\xi_{3}\left(t\right)}{p_{i}}\right] \end{array}$$

in the case of the influence of PK-SMC-self-other and PK-SMC-self-ecological.

In most of our simulations, we will use the simplest initial conditions: (i) at time t = 0, two agents are randomly distributed in space, (ii) they have the same absolute velocity κ, and (iii) they have randomly distributed directions θ. The directions {θ_*i*_} of the agents are determined simultaneously at each time step, and the position of the *i*−th agent is updated according to Equation (7). The value of parameter *L* (size of movement space) was taken equal to 5 for all shown simulations. For this value of *L*, the results shown here are valid for κ ∈ (0.001, 0.1), and we used κ = 0.05 for all graphics shown.

Our first main goal is to find the conditions under which the agents are capable of coordinating their movement (PK-SMC-self other). We measure the success of this simple task by calculating the average velocity *v*_*a*_ proposed in Vicsek et al. (1995) as follows:

(8) $$\begin{array}{l} v_{a}=\frac{1}{2\kappa }\left\|\overset{2}{\underset{i=1}{\sum }}{\text{v}}_{i}\right\| \end{array}$$

with **v**_*i*_ as the vector defined as **v**_*i*_ = κ(cos θ_*i*_. sin θ_*i*_) and ‖.‖ as the norm function. If *v*_*a*_ ≈ 1.0, we can say that our agents were capable of performing the task of coordinating successfully; if this is not the case, they failed it.

**Figure 5:**
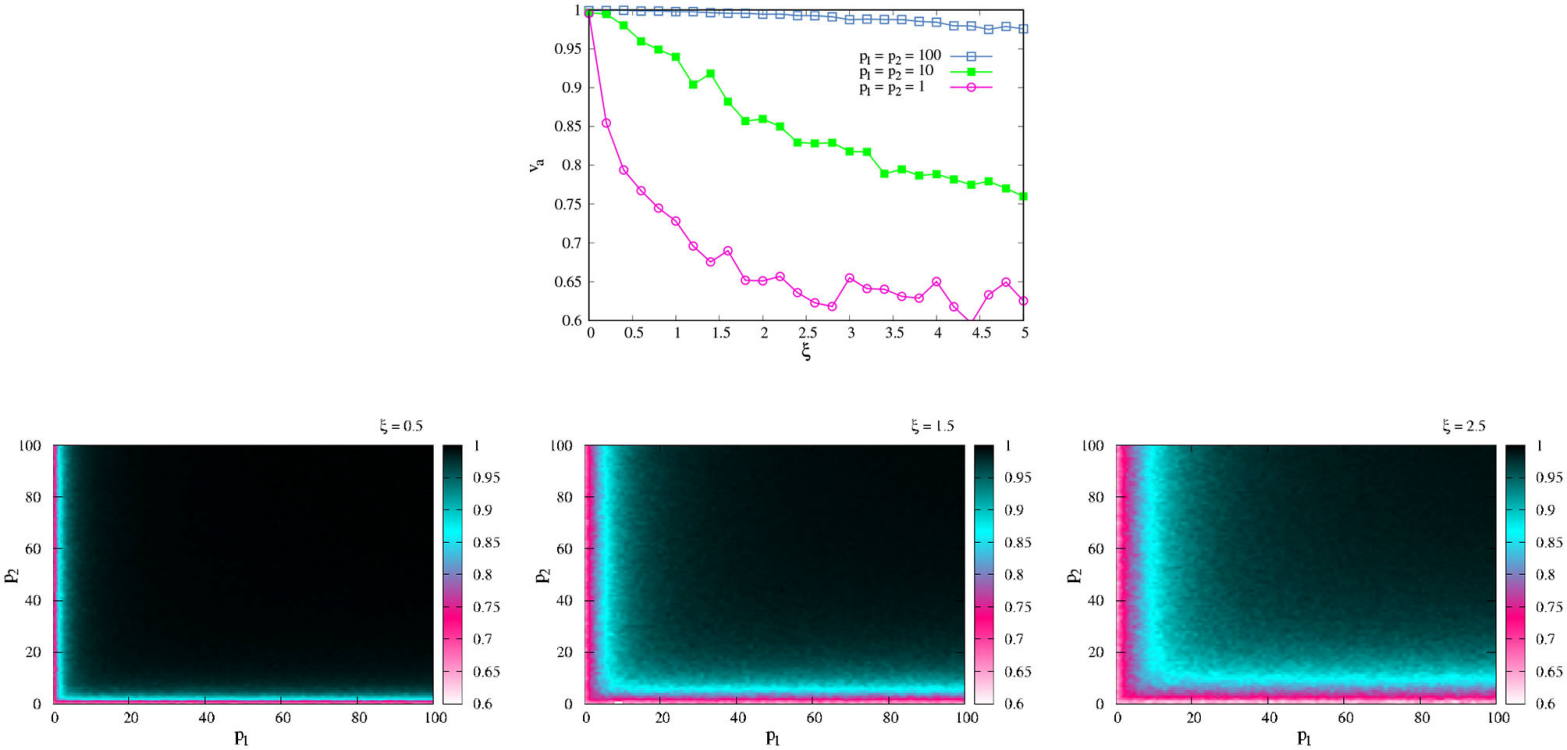
Effect of noise in coordination. **(Top)** *v*_*a*_ as function of ξ for different values of *p*. Here, we suppose that *p*_1_ = *p*_2_. **(Bottom)** *v*_*a*_ as function of (*p*_1_, *p*_2_) for different values of ξ. In all cases the graphs show the average 500 different experiments, each one with *t* = 2, 500.

The upper panel of Figure 5 shows the change of *v*_*a*_ as a function of ξ for different values of *p*. Here, we supposed that both agents have the same ability of PK, i.e., *p*_1_ = *p*_2_. We can see that values of ξ close to zero, even the lower values of *p* (= 1), achieved coordination. In another way, for larger values of ξ(> 3), even the agents with high PK *p* (= 100) are not able to coordinate their movement. Those values of ξ that are of interest are those in which 0.5 ≤ ξ ≤ 2.5, as in this range the effect of *p* is consistent with what we know about PK: individuals with high *p* (SA) are aware of their position in the world and recognize their possibilities for coordination.

The lower panels of Figure 5 shows the effect of noise in *v*_*a*_ as a function of (*p*_1_, *p*_2_). The different color maps show the combination of the values of *p*_*i*_ for which the agents are, or are not, coordinated. Here we can see that, for low values of noise (Figure 5, bottom left), the only values of *p*_*i*_ that impede a successful task are those that are really low (*p*_*i*_ ≤ 10). It is enough that one of the agents has this value of PK for coordination not to be reached regardless of whether the other agent has a very good value of *p*_*i*_ (pink and blue zones). On the contrary, if an agent with a PK that is not too low, or medium PK, interacts with an agent with high PK, both end up coordinating their movement (black zone). The effect of noise in decreasing PK values (v.g.r. Figure 5, up green curve) then disappears by the interaction with agents with better ability. The left two panels (Figure 5, low Center and Right) show similar results for higher values of ξ, and it is clear that if noise increases, the pink and blue zones in the color map are bigger, and larger values of *p*_*i*_ are necessary to achieve coordination. From here we will consider, in the rest of the results, ξ = 0.5, which is the value in which the impact of *p* is clearer.

Finally, we investigate the effect of *p*, α, and β not only on the ability of an isolated agent to find the center of concentration but on the ability of two agents to successfully interact with their environment and interact between them and to coordinate their movement (PK-SMC-self-ecological and PK-SMC-self-other). The task is to find in a coordinated way the center of concentration.

**Figure 6:**
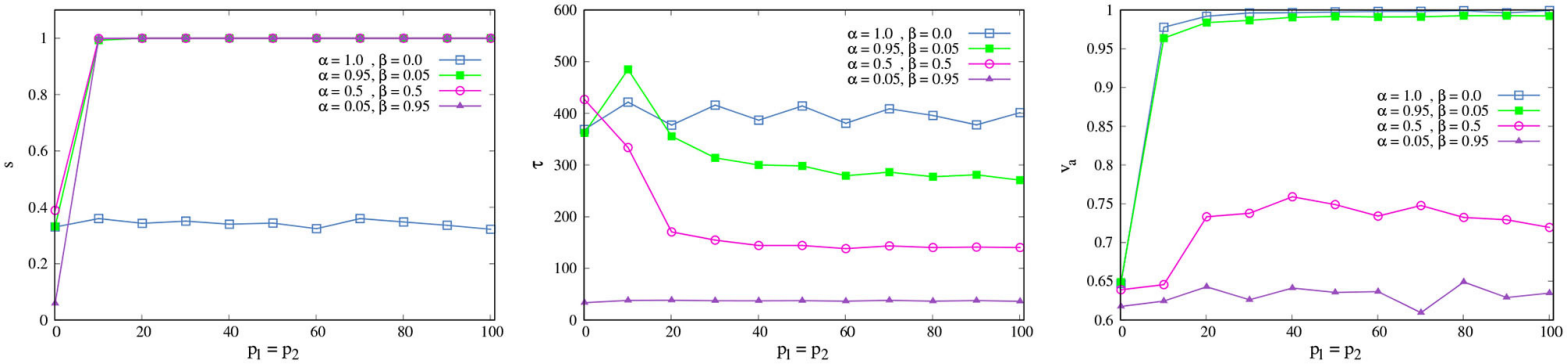
Environmental interaction. Average success rate *s* **(Left)**, average first-arrival time τ **(Middle)**, and average velocity *v*_*a*_ **(Right)** as functions of *p* (*p*_1_ = *p*_2_) for different combinations of α and β. In all cases, the graphs are the result of an average of 500 different experiments, each one with *t* = 2, 500.

Figure 6 shows the change of *s* (Left), τ (Center), and *v*_*a*_ (Right) as functions of *p* for different combinations of α, β. Here, we supposed that *p*_1_ = *p*_2_. We see that when α = 1 and β = 0 (blue squares), the agents are capable of coordinating for *p* ≥ 20. Their ability to always find the concentration center (*s* ≈ 0.4) is, however, very low, and when they can do it, they take a long time (τ ≈ 400). On the contrary, when α = β = 0.5 (pink circles) and α = 0.05 and β = 0.95 (purple triangles), the individuals with *p* ≥ 20 have a very good interaction with their environment; they can always find the point of greatest concentration (*s* = 1.0) and in a very short time (τ < 200), but they cannot coordinate their movement (*v*_*a*_ ≉ 1). Finally, when α = 0.95 and β = 0.05 (green squares), the agents are able to coordinate for *p* ≥ 40, and they can also quickly find the point of greatest concentration (*s* = 1.0 and τ < 400).

The above graphs show us that for medium values of PK *p* and α = 0.95, β = 0.05 our SPK agent can have a successful interaction with their environment and coordinating their movement. But to check if they can solve the task correctly, it is necessary to investigate if they arrive at the concentration center in a coordinated way.

**Figure 7:**
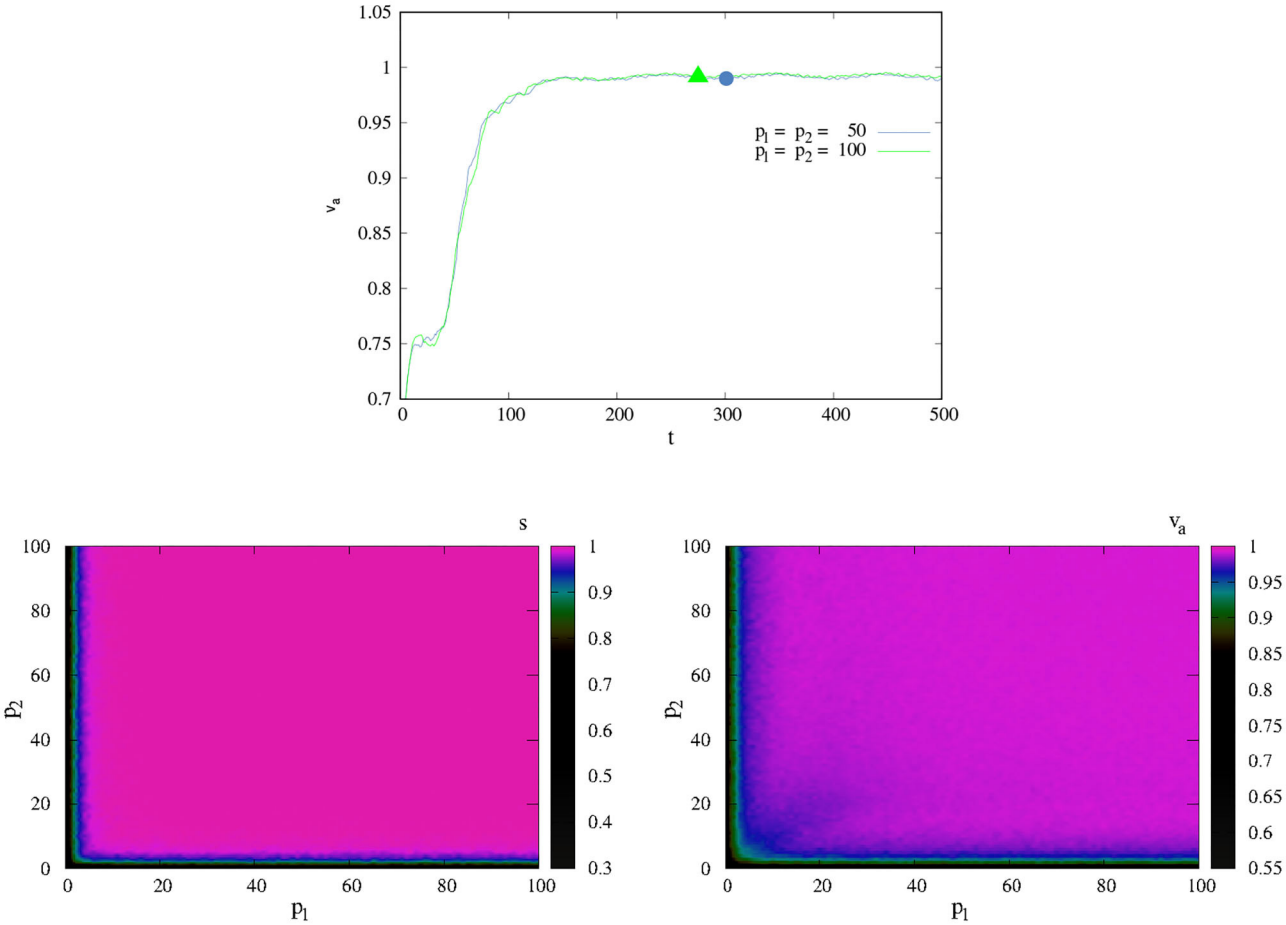
Achieving success in a task. **(Top)** *v*_*a*_ as function of *t* for different PK values *p*. Symbols represents the average first-arrival time for *p* = 50 (blue circle) and *p* = 100 (green triangle). **(Bottom)** *s* (Left) and *v*_*a*_ (Right) as function of (*p*_1_, *p*_2_). In all cases the graphs are the result of averaging 500 different experiments, each one with *t* = 2, 500.

Figure 7, top shows the change of *v*_*a*_ as a time increase for SPK agent with different values of PK (*p* = 50-blue line and *p* = 100-green line). We can see that for times >150, the agents are capable of coordination. The blue circle shows the average first-arrival time for agents with *p* equal to 50, and the green triangle portrays the same quantity but for *p* = 100. Both symbols lie in the section of the curve in which the agents are already coordinated. We can therefore say that for medium, or greater, values of PK (*p* ≥ 50), the agents are capable of solving the task successfully.

Figure 7, bottom shows *s* (Right) and *v*_*a*_ (Left) as functions of (*p*_1_, *p*_2_). For NSA (*p*_*i*_ ≤ 5), the success rate improves only with the interaction with an SA (pink zone). But for the task to be solved in coordination (*v*_*a*_ ≈ 1), it is necessary that one of the agents has a medium value of PK (*p*_*i*_ ≥ 40) and the other has the same or greater *p*. This means that an agent with high SPK improves the performance of an agent with lower SKP. The PK experience of both agents then arises from their own activity when interacting with others or through their self-other proprioception.

## Discussion

In this paper, we addressed the puzzle of proprioception in action from an ESMT and a phenomenological perspective. Arguing that PK coupling cannot be explained solely in terms of a body position sense or in mechanical terms about the pre-programming of the motor outcome, we proposed a theoretical and formal framework to understand how the PK perceptual experience is a form of mastering and dynamical learning about body orientation, possibilities for action, and felt qualitative dynamics. This allows us to take into consideration two missing dimensions in current accounts of proprioceptive perception in action: self-ecological and self-other relationships and felt experiences. Recognizing this type of relational nature has epistemological implications that can encourage deep research in these issues.

While ESMT has been mostly developed for the visual and tactile modalities, we believe that the arguments and evidence in favor of ESMT should generalize to other perceptive modalities (Lyon, 2014). Here, we have focused on applying this theory to the PK modality. We have presented a minimal model to describe PK-SMCs, which assumes that the perceptual skill or ability of proprioception/kinesthesia is described by a single parameter *p*. The main model equations portray the fact that proprioception is coupled with kinesthesia, i.e., a proprioceptive agent senses her body and performs it.

Our results showed that NSPK (low *p*) are not capable of making a distinction between the three sources of variation in the PK sensory signals:

– *PK-SMCs-self:* They cannot recognize their own position in the world, and their movement in it is erratic. This is an immediate consequence of the structure of equations that define the agent's position and movement.
– *PK-SMCs-self-ecological:* Because the NSPK agent are not able to recognize their own position in the world and, therefore, are not capable of moving in it correctly, their interaction with the environment is poor, and they are not capable of recognizing the different signals that come from it. It is impossible for them to solve the task of finding the center of a concentration gradient efficiently.
– *PK-SMCs-self-other:* The impossibility of NSPK agent to recognize their position in the world leads to an impossibility of interacting with another agent. The NSPK is not capable of sensing whether the other is (or is not) inside of their interaction radius.

On other hand, SPK agent (high *p*) are perfectly capable of making distinctions between the three different sources of variation in PK sensory signals mentioned above. Furthermore, they are capable of solving tasks in coordination with the other, the environment, and both the other and the environment. The PK experience of this kind of agents is constituted by the three PK-SMCs: those that are related to their own orientation and action possibilities in present time or self -proprioception (PK-self); those that arise from their own activity when interacting with the environment or self-ecological-proprioception (PK-self-environment); and those that arise from their own activity when interacting with others or self-other-proprioception (PK-self-other).

A remarkable result is that the agents with medium values of *p* can make a better distinction between PK-SMs-self, self-ecological, and self-other if they interact with agents with higher values of PK. Interaction helps to improve the performance of the agents. Then, the unit of analysis of ecological and dyadic interaction,—as a minimal form of ecological and social cognition—is thus no longer reduced to the individual, but makes reference to a system as a (self-)organized whole, including the agents involved in the interaction, the process of interaction itself, as well as the ecological context in which these interactions take place.

Despite the minimal PK model's simplicity (or rather thanks to it), this finding might be a good starting point for formalizing Merleau-Ponty's statement that when perceiving others “there exists an internal relation that causes the other to appear as the completion of the system” (Merleau-Ponty, 1945, p. 410). This is because the maintenance of the coordinated behavior, which can take place in two distinct regions of state space depending on whether the agents are jointly moving leftward or rightward, depends on the active participation of the other agent. The proposed distinctions are part of the theoretical and formal approach, but, in reality, these three sources of variation are always intertwined due to felt experiences, perception, and learning, which are ongoing and dynamical processes that in many senses are impossible to consider as separate.

Furthermore, our model shows that this significant increase in the preference for the other agent (with whom it is easiest to coordinate) cannot be explained satisfactorily in terms of only the individual's cognitive assessment of the other's presence: it also requires us to take into account the level of relations between the interactants, as reflected by their capacity for joint contingency recognition and the synchronized timing of their respective assessments. We demonstrate this to be the case in our PK minimal model and thus challenge methodological individualism, as have Kelso et al. (2013)'s coupled dynamical systems and Auvray et al. (2009)'s interactionist account perspectives.

This minimal agent-based model therefore serves as a formal proof of concept that the learning or mastering of skills related to the PK-SMCs-self, PK-SMCs-self-environment, and PK-SMCs self-other, such as when two agents reciprocally participate in the interactive realization of each other's socially contingent actions, is possible in principle. Perhaps in the near future, these findings can also be empirically confirmed in actual psychological experiments of social interaction—in particular those that also take into account the sensorimotor conscious experience of the participants.

In sum, this model is simple and summarizes in a few parameters several mechanisms and actions that could be specified in more explicit ways in a more realistic version. On the other hand, we interpreted the parameters α and β as the capability of an acting agent to make a distinction between the sensory source of her own movement and the sensory source that comes from her interaction with the environment. These are free parameters, and they were adjusted so that the agents could solve a particular task. A possible extension of this minimal PK model would be to consider these parameters α and β as functions of *p*, which would imply that the capability of an agent to perceive these two kinds of movement sources depended on her ability of PK. Finally, in order to portray the fact that the PK experience is an ability that can be learned (and improved) through experience, a future extension could be that the parameter *p* changed as a function of time and different kinds of interactions (social and ecological).

A small but growing number of experimental, psychological, and simulation studies have investigated the constitutive role of the ecological and social interaction for proprioception or for social cognition. Ecological studies about the dynamic touch have begun to produce interesting data. For instance, Asao et al. (2012) demonstrated experimentally that proprioception is important for perceiving the length only through identifying physical invariants and potential movements. In addition, research based on the perceptual crossing paradigm has also contributed to this kind of development. With this aim, Auvray and Rohde (2012) predicts that the acquisition of the ability to detect the responsive presence of others is an embodied skill that goes together with a measurable change in the agent's experience.

However, the potential link between evidence of PK coupling, ESMT, and social interaction is still in need of further development to strengthen its epistemological implications, both because the ESMT of proprioception requires clarification and because its neurophysiological and neuroscientific predictions must be made still more explicit.

## Conclusions

This research prompts us to think not only in reflective terms when we refer to a skilled perceptual PK experience but also on the attentive learning of PK-SMCs and particular kinds of feelings or sensibilities. Nevertheless, from the weak embodiment perspective, it is complicated to extend the neural representation toward peripheral, autonomic, ecological, and social aspects of embodiment. The perspective that we have defended here is a stronger notion of embodiment. We suggest that it is the PK system, with its coupling history of interacting and by the individual's personal experiences, that enables specific perception-action loops, learning to interact and to respond to the world rather than representing it. Specifically, skilled proprioceptive and kinesthetic coupling plays an important role in the felt perceptual experience of spatio-temporal self-orientation in present action and interaction in ways that are irreducible to B-formatted representations.

In our proposed minimal model, the PK perceptual experience of the agents is constituted by three PK-SMCs that are related to its own orientation and action possibilities in present time or self-PK (PK-self); those that arise from its own activity when interacting with the environment or self-ecological-PK (PK-self-environment); and those that arise from its own activity when interacting with others or self-other-proprioception (PK-self-other). Besides helping us to differentiate between NSPK and SPK agent, the model provides important results, including the fact that interaction helps to improve the performance of the agents. This finding might be a good starting point for formalizing the statements of interactions discussed by Merleau-Ponty (1945), Kelso et al. (2013), and Auvray et al. (2009). This minimal agent-based model therefore serves as a formal proof of concept that the learning or mastering of skills related to the different PK-SMCs is possible.

In this sense, PK perceptual experience crystallizes as a specific type of coordination of the organism's action with opportunities afforded by the self, the self-environment, and the self-other-environment. In other words, it is necessary to consider the specific organism-environment interactions that the living process would engage in, tracing the path to overcome the transition from subpersonal representations to personal experience. These abilities are meaningful because the agent has learned them through a history of perception and action coupling and does not require internal comparison models.

We think that the strong embodiment strategy used in this paper, contributes to closing the gap between the content of the proprioceptive-kinesthetic perceptual experience and the skilled possibilities for action.

## Author Contributions

XG-G provided the original idea. AF-C and GR-F developed the agent-based model. All authors discussed the general outline, the theoretical framework of the article, and contributed to comments and revisions.

## Conflict of Interest

The authors declare that the research was conducted in the absence of any commercial or financial relationships that could be construed as a potential conflict of interest.

## Footnotes

^1^A general description of proprioceptive feedback (PF) as an integral component of vertebrate locomotor control (Prochazka and Ellaway, 2012; Gordon et al., 2019) would be the result of two different processes: self-generated reflexes from nervous pathways to each muscle via spinal interneurons regulating the ongoing activity and mechanical output of multiple muscles, and longer-latency pathways to spinal networks and higher CNS areas (cerebellum, basal ganglia, and cortex). Both processes are important to estimate state and update internal models to coordinate balance and plan movement (Wolpert et al., 1995; Wolpert and Ghahramani, 2000; Proske and Gandevia, 2012).

^2^Furthermore, neurophysiological research has shown that brain area activations during passive and active proprioception, as somatosensory evoked potentials (SEP), seem to be both afferent (due to the activation of peripheral afferents) and efferent (the influences of descending pyramidal and extrapyramidal influences; Coquery et al., 1972; Beets et al., 2012).

^3^The internal models for motor control theories—neural mechanisms that can mimic the afferent/efferent characteristics or their inverses—assume that the central nervous system (CNS) is able to prepare in advance to differentiate between these two classes of sensory afferences, by sending a parallel “efference copy” of its motor command to sensory areas (von Holst E and Mittelstaedt, 1950; Kawato, 1999).

^4^Feldman (2011) proposes the equilibrium-point theory in response to the posture-movement problem: why active movements away from a stable posture are not opposed by stabilizing mechanisms, rather than being specific neural structures representing spatial frames of reference selected by the brain.

^5^This theory is often considered part of 4E Cognition perspectives in cognitive science, where cognition is embodied, enactive, ecological, and extended. However, the heterogeneity of the different lines of research within 4EC has led to certain disagreements that have partially split their bond. It is not the purpose of this article to delve into those disagreements, and for our purposes, we will consider that they share these basic claims, and, therefore, we can raise them as bonded (Gonzalez-Grandón and Froese, 2018): (a) cognition depends on the characteristics of the agent's body and its interaction with the physical and social environment, and (b) the rejection of mainstream theories, which treats proprioception, for instance, as a passive process of sensorimotor information processing in the brain to set up detailed internal mental representations of the body and its parts (Bermúdez, 2000; Gallagher, 2003; Cardinali et al., 2009).

^6^Some theories of the sense of bodily ownership try to address how an agent goes from a proprioceptive experience with the non-conceptual content to a proprioceptive judgment with the conceptual content (Peacocke, 2014). In this sense, (Gallagher, 2003, p. 3) contrasts the typical and “non-reflective awareness of the body,” with proprioceptive awareness as an introspective or reflective type of proprioception. However, we think that we do not need to overly intellectualize human embodied experiences in order to classify them as genuinely perceptual.

^7^Our PK phenomenology proposal has a lot of assumptions that are by no means universally accepted. We limit ourselves to highlight that we ought to consider alternative methods for understanding and distinguishing the nature of such PK experience and their relation to our proposal. On our side, we think we ought to rely on both first and third-person perspectives—phenomenological descriptions of experience contrasted with naturalistic explanations—in order to come up with accurate and useful categorizations of these conscious states. Such a dialogue platform will be more likely to yield a solid theoretical PK experience foundation.

^8^Since we do not have enough prior information about the behavior of this external and internal stimulus that could modify the agent's perception, the adequate distribution to portray them is a uniform one.

^9^The variables ξ_1_ are sampled at every time because of these “other factors” influence in the agent's movement at each time step of the dynamics.

^10^The uniform interval takes negative values only because the 2*d* square environment has negative coordinates.

^11^In general ξ_1_(*t*) ≠ ξ_2_(*t*). This is because the things that could change the agent's perception of their position in the world are not always the same as the things that could change the agent's perception of their possibilities for movement.

^12^The large jumps are due to the fact that the space has periodic boundaries: when one coordinate (*x* or *y*) in the agent's positions is too close to the boundary, it appears in the other side of the space.

^13^Some authors interested in the factors that contribute to the sense of position have reported that position acuity may be improved by increasing the activity of the musculotendinous receptors, for example, by a loaded limb condition (Suprak et al., 2007).

^14^Furthermore, it seems to be following an idea closer to weak embodiment, to B-format notion, where the updating takes place only at an internal level, without requiring an attentive effort in some steps of the learning process.

^15^It is not the goal of this article to go into depth in the consideration of many detailed levels of awareness when interacting in different socio-cultural practices. However, it seems certain that learning about particularities of daily life that develop relatively stable patterns of coordination toward a specific practical mode (for the kind of work we do or for games we play) may lead to different levels of PK awareness.

^16^In this sense, Sheets-Johnstone (2019) propose that from the first social interactions (e.g., newborn-caregiver), the agent incorporates the dynamic flow of body proprioceptive and kinesthetic signals (PK-SMCs of gestures, gaze, gait, etc.) from others into how they modulate their own actions.

^17^“From an enactive viewpoint, other bodies dynamically interpenetrate our own bodily actions and thereby provide a flux of resources for orienting our actions” (Kimmel, 2013, p. 313).

## References

[B1] Alaerts K. Levin O. Swinnen S. P. (2007). Whether feeling or seeing is more accurate depends on tracking direction within the perception-action cycle. Behav. Brain Res. 178, 229–234. 10.1016/j.bbr.2006.12.024 17280724

[B2] Alsmith A. J. T. De Vignemont F. (2012). Embodying the mind and representing the body. Rev. Philos. Psychol. 3, 1–13. 10.1007/s13164-012-0085-4

[B3] Asao T. Suzuki S. Kotani K. (2012). Mechanism of length perception by dynamic touch-proposal of identification-perception model considering proprioception, in 2012 ICME International Conference on Complex Medical Engineering (CME) (Kobe), 402–407. 10.1109/ICCME.2012.6275734

[B4] Asatryan D. Feldman A. (1965). Biophysics of complex systems and mathematical models. functional tuning of nervous system with control of movement or maintenance of a steady posture. I. Mechanographic analysis of the work of the joint on execution of a postural task. Biophysics 10, 925–935.

[B5] Auvray M. Lenay C. Stewart J. (2009). Perceptual interactions in a minimalist virtual environment. New Ideas Psychol. 27, 32–47. 10.1016/j.newideapsych.2007.12.002

[B6] Auvray M. Rohde M. (2012). Perceptual crossing: the simplest online paradigm. Front. Hum. Neurosci. 6:181. 10.3389/fnhum.2012.00181 22723776 PMC3377933

[B7] Beer R. D. (2003). The dynamics of active categorical perception in an evolved model agent. Adapt. Behav. 11, 209–243. 10.1177/105971230311400119362447

[B8] Beets I. A. Macé M. Meesen R. L. Cuypers K. Levin O. Swinnen S. P. (2012). Active versus passive training of a complex bimanual task: is prescriptive proprioceptive information sufficient for inducing motor learning? PLoS ONE 7:e37687. 10.1371/journal.pone.0037687 22666379 PMC3359339

[B9] Bermúdez J. L. (2000). The Paradox of Self-Consciousness. Cambridge: MIT Press.

[B10] Botvinick M. Cohen J. (1998). Rubber hands-feel-touch that eyes see. Nature 391, 756–756. 10.1038/35784 9486643

[B11] Buhrmann T. Di Paolo E. A. Barandiaran X. (2013). A dynamical systems account of sensorimotor contingencies. Front. Psychol. 4:285. 10.3389/fpsyg.2013.00285 23750143 PMC3664438

[B12] Capaday C. Darling W. G. Stanek K. Van Vreeswijk C. (2013). Pointing to oneself: active versus passive proprioception revisited and implications for internal models of motor system function. Exp. Brain Res. 229, 171–180. 10.1007/s00221-013-3603-4 23756602

[B13] Cardinali L. Brozzoli C. Farne A. (2009). Peripersonal space and body schema: two labels for the same concept? Brain Topogr. 21, 252–260. 10.1007/s10548-009-0092-7 19387818

[B14] Connell L. A. Lincoln N. Radford K. (2008). Somatosensory impairment after stroke: frequency of different deficits and their recovery. Clin. Rehabil. 22, 758–767. 10.1177/0269215508090674 18678576

[B15] Coquery J.-M. Coulmance M. Leron M.-C. (1972). Modifications des potentiels évoqués corticaux somesthésiques durant des mouvements actifs et passifs chez l'homme. Electroencephalogr. Clin. Neurophysiol. 33, 269–276. 10.1016/0013-4694(72)90153-8 4114910

[B16] Craggs M. Rothwell J. Rushton D. (1979). Gating of somatosensory evoked potentials by active and passive movements in man [proceedings]. J. Physiol. 295:96P. 522007

[B17] Crane T. (1992). The Contents of Experience: Essays on Perception. Cambridge: Cambridge University Press 10.1017/CBO9780511554582

[B18] Crapse T. B. Sommer M. A. (2008). Corollary discharge circuits in the primate brain. Curr. Opin. Neurobiol. 18, 552–557. 10.1016/j.conb.2008.09.017 18848626 PMC2702467

[B19] Darling W. G. Wall B. M. Coffman C. R. Capaday C. (2018). Pointing to one's moving hand: Putative internal models do not contribute to proprioceptive acuity. Front. Hum. Neurosci. 12:177. 10.3389/fnhum.2018.00177 29867407 PMC5962794

[B20] Dayan E. Casile A. Levit-Binnun N. Giese M. A. Hendler T. Flash T. (2007). Neural representations of kinematic laws of motion: evidence for action-perception coupling. Proc. Natl. Acad. Sci. U.S.A. 104, 20582–20587. 10.1073/pnas.0710033104 18079289 PMC2154474

[B21] De Jaegher H. Di Paolo E. Gallagher S. (2010). Can social interaction constitute social cognition? Trends Cogn. Sci. 14, 441–447. 10.1016/j.tics.2010.06.009 20674467

[B22] De Vignemont F. (2018). Mind the Body: An Exploration of Bodily Self-Awareness. Oxford: Oxford University Press 10.1093/oso/9780198735885.001.0001

[B23] Doeden M. (2014). Basketball Legends in the Making. Minnesota: Capstone.

[B24] Dreyfus H. L. (1996). The current relevance of merleau-ponty's phenomenology of embodiment. Electron. J. Analyt. Philos. 4, 1–16.

[B25] Eklund G. (1972). General features of vibration-induced effects on balance. Upsala J. Med. Sci. 77, 112–124. 10.1517/03009734000000016 4262735

[B26] Farrer C. Franck N. Paillard J. Jeannerod M. (2003). The role of proprioception in action recognition. Conscious. Cogn. 12, 609–619. 10.1016/S1053-8100(03)00047-3 14656504

[B27] Feldman A. G. (2011). Space and time in the context of equilibrium-point theory. Wiley Interdiscip. Rev. 2, 287–304. 10.1002/wcs.108 26302077

[B28] Feldman A. G. (2016). The relationship between postural and movement stability, in Progress in Motor Control, eds Laczko J. Latash M. (Cham: Springer), 105–120. 10.1007/978-3-319-47313-0_6

[B29] Fourneret P. Jeannerod M. (1998). Limited conscious monitoring of motor performance in normal subjects. Neuropsychologia 36, 1133–1140. 10.1016/S0028-3932(98)00006-2 9842759

[B30] Fridland E. (2011). The case for proprioception. Phenomenol. Cogn. Sci. 10:521 10.1007/s11097-011-9217-z

[B31] Froese T. González-Grandón X. (2019). How passive is passive listening? Toward a sensorimotor theory of auditory perception. Phenomenol. Cogn. Sci. 1–33.

[B32] Froese T. Zapata-Fonseca L. Leenen I. Fossion R. (2020). The feeling is mutual: clarity of haptics-mediated social perception is not associated with the recognition of the other, only with recognition of each other. Front. Hum. Neurosci. 14:560567. 10.3389/fnhum.2020.560567 33088267 PMC7500513

[B33] Gallagher S. (2003). Bodily self-awareness and object perception. Theor. Hist. Sci. 7, 53–68. 10.12775/ths.2003.004

[B34] Gallagher S. (2006). How the Body Shapes the Mind. Oxford: Clarendon Press 10.1093/0199271941.001.0001

[B35] Gallagher S. (2009). Deep and dynamic interaction: response to hanne de jaegher. Conscious. Cogn. 18, 547–548. 10.1016/j.concog.2008.12.010

[B36] Gallagher S. (2017). Self-defense: deflecting deflationary and eliminativist critiques of the sense of ownership. Front. Psychol. 8:1612. 10.3389/fpsyg.2017.01612 28970813 PMC5609435

[B37] Gallagher S. Zahavi D. (2012). The Phenomenological Mind. Abingdon, VA: Routledge 10.4324/9780203126752

[B38] Gapenne O. (2010). Kinesthesia and the construction of perceptual objects, in Enaction: Toward a New Paradigm for Cognitive Science, eds Stewart J. Gapenne O. Di Paolo E. A. (Cambridge: MIT Press), 183–218. 10.7551/mitpress/9780262014601.003.0008

[B39] Gapenne O. (2014). The co-constitution of the self and the world: action and proprioceptive coupling. Front. Psychol. 5:594. 10.3389/fpsyg.2014.00594 24971073 PMC4054590

[B40] Gibson J. (1977). The concept of affordances. Perceiv. Acting Know. 1, 67–82.

[B41] Goldman A. de Vignemont F. (2009). Is social cognition embodied? Trends Cogn. Sci. 13, 154–159. 10.1016/j.tics.2009.01.007 19269881

[B42] Goldman A. I. (2012). A moderate approach to embodied cognitive science. Rev. Philos. Psychol. 3, 71–88. 10.1007/s13164-012-0089-0

[B43] Gonzalez-Grandón X. Froese T. (2018). Grounding 4E cognition in Mexico: introduction to special issue on spotlight on 4E cognition research in Mexico. SAGE 26, 189–198. 10.1177/1059712318791633

[B44] Goodwin G. McCloskey D. Matthews P. (1972). The contribution of muscle afferents to kinesthesia shown by vibration induced illusions of movement and by the effects of paralysing joint afferents. Brain 95, 705–748. 10.1093/brain/95.4.705 4265060

[B45] Gordon J. C. Holt N. C. Biewener A. A. Daley M. A. (2019). Tuning of feedforward control enables stable muscle force length dynamics after loss of autogenic proprioceptive feedback. eLife 9:e53908. 10.7554/eLife.53908 32573432 PMC7334023

[B46] Henri P. (1902). La Science et l'hypothése. Paris: Flammarion.

[B47] Hewett T. E. Paterno M. V. Myer G. D. (2002). Strategies for enhancing proprioception and neuromuscular control of the knee. Clin. Orthop. Relat. Res. 402, 76–94. 10.1097/00003086-200209000-00008 12218474

[B48] Howe K. A. (2018). Proprioceptive awareness and practical unity. Teorema 37, 65–82.

[B49] Husserl E. (1989). Ideas Pertaining to a Pure Phenomenology and to a Phenomenological Philosophy: Second Book Studies in the Phenomenology of Constitution, Vol. 3. Dordrecht: Springer Science & Business Media 10.1007/978-94-009-2233-4

[B50] Ilmane N. Sangani S. Feldman A. G. (2013). Corticospinal control strategies underlying voluntary and involuntary wrist movements. Behav. Brain Res. 236, 350–358. 10.1016/j.bbr.2012.09.008 22983216

[B51] Iscla I. Blount P. (2012). Sensing and responding to membrane tension: the bacterial MSCL channel as a model system. Biophys. J. 103, 169–174. 10.1016/j.bpj.2012.06.021 22853893 PMC3400780

[B52] Kawato M. (1999). Internal models for motor control and trajectory planning. Curr. Opin. Neurobiol. 9, 718–727. 10.1016/S0959-4388(99)00028-8 10607637

[B53] Kelso J. Dumas G. Tognoli E. (2013). Outline of a general theory of behavior and brain coordination. Neural Netw. 37, 120–131. 10.1016/j.neunet.2012.09.003 23084845 PMC3914303

[B54] Kiefer A. W. Riley M. A. Shockley K. Sitton C. A. Hewett T. E. Cummins-Sebree S.. (2013). Lower-limb proprioceptive awareness in professional ballet dancers. J. Dance Med. Sci. 17, 126–132. 10.12678/1089-313X.17.3.126 24069947

[B55] Kimmel M. (2013). The arc from the body to culture: how affect, proprioception, kinesthesia, and perceptual imagery shape cultural knowledge (and vice versa). Integr. Rev. 9:300–348.

[B56] Lackner J. R. (1988). Some proprioceptive influences on the perceptual representation of body shape and orientation. Brain 111, 281–297. 10.1093/brain/111.2.281 3378137

[B57] Lebois F. Sauvage P. Py C. Cardoso O. Ladoux B. Hersen P. Di Meglio J.-M. (2012). Locomotion control of caenorhabditis elegans through confinement. Biophys. J. 102, 2791–2798. 10.1016/j.bpj.2012.04.051 22735529 PMC3379027

[B58] Lenay C. (2006). Enaction, externalisme et suppléance perceptive. Intellectica 43, 27–52. 10.3406/intel.2006.1326

[B59] Lyon C. (2014). Beyond vision: extending the scope of a sensorimotor account of perception, in Contemporary Sensorimotor Theory, eds Bishop J. Martin A. (Cham: Springer), 127–136. 10.1007/978-3-319-05107-9_9

[B60] McGann M. De Jaegher H. (2009). Self-other contingencies: enacting social perception. Phenomenol. Cogn. Sci. 8, 417–437. 10.1007/s11097-009-9141-7

[B61] Merleau-Ponty M. (1945). Phénoménologie de la Perception. Paris: Gallimard.

[B62] Mitsuo K. Tomoe K. Hiroshi I. Eri N. Satoru M. Toshinori Y. (2003). Internal forward models in the cerebellum: fMRI study on grip force and load force coupling. Prog. Brain Res. 142, 171–188. 10.1016/S0079-6123(03)42013-X 12693261

[B63] Myin E. (2016). Perception as something we do. J. Conscious. Stud. 23, 80–104.

[B64] Myin E. O'Regan J. K. (2002). Perceptual consciousness, access to modality and skill theories. a way to naturalize phenomenology? J. Conscious. Stud. 9, 27–46.

[B65] Nakajima T. Wasaka T. Kida T. Nishimura Y. Fumoto M. Sakamoto M.. (2006). Changes in somatosensory evoked potentials and Hoffmann reflexes during fast isometric contraction of foot plantarflexor in humans. Percept. Motor Skills 103, 847–860. 10.2466/pms.103.3.847-860 17326514

[B66] Neisser U. (1988). Five kinds of self-knowledge. Philos. Psychol. 1, 35–59. 10.1080/09515088808572924

[B67] Noë A. (2002). Is the visual world a grand illusion? J. Conscious. Stud. 9, 1–12.

[B68] Noë A. (2004). Action in Perception. Cambridge: MIT Press.

[B69] Noë A. O'Regan J. K. (2000). Perception, attention, and the grand illusion. Psyche 6, 6–15.

[B70] O'Regan J. (2011). Why Red Doesn't Sound Like A Bell: Understanding the Feel of Consciousness. Oxford: Oxford University Press 10.1093/acprof:oso/9780199775224.001.0001

[B71] O'Regan J. K. Myin E. Noë A. (2004). Towards an analytic phenomenology: the concepts of “bodiliness” and “grabbiness”, in Seeing, Thinking and Knowing, ed Carsetti A. (Dordrecht: Springer), 103–114. 10.1007/1-4020-2081-3_5

[B72] O'Regan J. K. Myin E. Noë A. (2005). Skill, corporality and alerting capacity in an account of sensory consciousness. Prog. Brain Res. 150, 55–592. 10.1016/S0079-6123(05)50005-0 16186015

[B73] O'Regan J. K. Noë A. (2001). A sensorimotor account of vision and visual consciousness. Behav. Brain Sci. 24, 939–973. 10.1017/S0140525X01000115 12239892

[B74] O'Regan J. K. Noe A. (2002). The origin of “feel”, in From Animals to Animats (Cambridge) Vol. 7, 27-35.

[B75] O'Shaughnessy B. (1995). Proprioception and the body image, in The Body and the Self, eds Bermúdez J. L. Mercel A. Eilan N. (Cambridge: MIT Press) 175–203.

[B76] O'shaughnessy B. (2008). The Will: Vol. 1, Dual Aspect Theory. Cambridge: Cambridge University Press.

[B77] Peacocke C. (2014). The Mirror of the World: Subjects, Consciousness, and Self-Consciousness. Oxford: Oxford University Press 10.1093/acprof:oso/9780199699568.001.0001

[B78] Philipona D. O'Regan J. Nadal J. (2003). Is there something out there? Inferring space from sensorimotor dependencies. Neural Comput. 15, 2029–2049. 10.1162/089976603322297278 12959664

[B79] Piaget J. (1937). La Construction du réel Chez l'enfant. París: Delachaux & Niestle.

[B80] Prochazka A. Ellaway P. (2012). Sensory systems in the control of movement. Comprehens. Physiol. 2, 2615–2627. 10.1002/cphy.c100086 23720260

[B81] Prosk,e U. Gandevia S. C. (2012). The proprioceptive senses: their roles in signaling body shape, body position and movement, and muscle force. Physiol. Rev. 92, 1651–1697. 10.1152/physrev.00048.2011 23073629

[B82] Sheets-Johnstone M. (2019). Kinesthesia: an extended critical overview and a beginning phenomenology of learning. Contin. Philos. Rev. 52, 143–169. 10.1007/s11007-018-09460-7

[B83] Sheets-Johnstone M. (2020). The lived body. Human. Psychol. 48:28 10.1037/hum0000150

[B84] Sherrington C. S. (1907). On the proprio-ceptive system, especially in its reflex aspect. Brain 29, 467–482. 10.1093/brain/29.4.467

[B85] Sherrington C. S. (1918). Observations on the sensual role of the proprioceptive nerve-supply of the extrinsic ocular muscles. Brain 41, 332–343. 10.1093/brain/41.3-4.332

[B86] Silverman D. (2018). Bodily skill and internal representation in sensorimotor perception. Phenomenol. Cogn. Sci. 17, 157–173. 10.1007/s11097-017-9503-5

[B87] Suprak D. N. Osternig L. R. van Donkelaar P. Karduna A. R. (2007). Shoulder joint position sense improves with external load. J. Motor Behav. 39, 517–525. 10.3200/JMBR.39.6.517-525 18055357

[B88] Sydney S. (1996). Self-knowledge and inner sense, in: Philosophy and Phenomenological Research LV I, 249–314.

[B89] Thelen E. (1990). Coupling perception and action in the development of skill: a dynamic approach, in Sensory-Motor Organizations and Development in Infancy and Early Childhood, eds Bloch H. Bertenthal B. I. (Dordrecht: Springer), 39–56. 10.1007/978-94-009-2071-2_3

[B90] Tsakiris M. (2015). The relations between agency and body ownership, in The Sense of Agency, P. Haggard and B. Eitam (Oxford: Oxford University Press), 235–256. 10.1093/acprof:oso/9780190267278.003.0010

[B91] Varela F. J. (1999). Present-time consciousness. J. Conscious. Stud. 6, 111–140.

[B92] Varela F. J. Thompson E. Rosch E. (1991). The Embodied Mind: Cognitive Science and Human Experience. Cambridge: MIT Press 10.7551/mitpress/6730.001.0001

[B93] Vicsek T. Czirók A. Ben-Jacob E. Cohen I. Shochet O. (1995). Novel type of phase transition in a system of self-driven particles. Phys. Rev. Lett. 75:1226. 10.1103/PhysRevLett.75.1226 10060237

[B94] von Holst E. Mittelstaedt H. (1950). Das reafferenzprinzip. Naturwissenschaften 37, 464–476. 10.1007/BF00622503

[B95] Warren W. (2006). The dynamics of perception and action. Psychol. Rev. 113, 358–389. 10.1037/0033-295X.113.2.358 16637765

[B96] Wolpert D. M. Diedrichsen J. Randall F. (2011). Principles of sensorimotor learning. Nat. Rev. Neurosci. 12, 739–751. 10.1038/nrn3112 22033537

[B97] Wolpert D. M. Ghahramani Z. (2000). Computational principles of movement neuroscience. Nat. Neurosci. 3, 1212–1217. 10.1038/81497 11127840

[B98] Wolpert D. M. Ghahramani Z. Jordan M. I. (1995). An internal model for sensorimotor integration. Science 269, 1880–1882. 10.1126/science.7569931 7569931

